# Annexin A2 combined with TTK accelerates esophageal cancer progression via the Akt/mTOR signaling pathway

**DOI:** 10.1038/s41419-024-06683-w

**Published:** 2024-04-24

**Authors:** Ruiqi Liu, Yanwei Lu, Jing Li, Weiping Yao, Jiajun Wu, Xiaoyan Chen, Luanluan Huang, Ding Nan, Yitian Zhang, Weijun Chen, Ying Wang, Yongshi Jia, Jianming Tang, Xiaodong Liang, Haibo Zhang

**Affiliations:** 1 Cancer Center, Department of Radiation Oncology, Zhejiang Provincial People’s Hospital (Affiliated People’s Hospital), Hangzhou Medical College, Hangzhou, Zhejiang China; 2 Graduate Department, Bengbu Medical College, Bengbu, Anhui China; 3 The First School of Clinical Medicine, Lanzhou University, Lanzhou, Gansu China; 4 Graduate Department, Hangzhou Normal University, Hangzhou, Zhejiang China; 5 Department of Oncology, Jinxiang People’s Hospital, Jining, Shandong China; 6 Department of Radiation Oncology, The First Hospital of Lanzhou University, Lanzhou University, Lanzhou, Gansu China

**Keywords:** Tumour biomarkers, Cancer therapy

## Abstract

Annexin A2 (ANXA2) is a widely reported oncogene. However, the mechanism of ANXA2 in esophageal cancer is not fully understood. In this study, we provided evidence that ANXA2 promotes the progression of esophageal squamous cell carcinoma (ESCC) through the downstream target threonine tyrosine kinase (TTK). These results are consistent with the up-regulation of ANXA2 and TTK in ESCC. In vitro experiments by knockdown and overexpression of ANXA2 revealed that ANXA2 promotes the progression of ESCC by enhancing cancer cell proliferation, migration, and invasion. Subsequently, animal models also confirmed the role of ANXA2 in promoting the proliferation and metastasis of ESCC. Mechanistically, the ANXA2/TTK complex activates the Akt/mTOR signaling pathway and accelerates epithelial-mesenchymal transition (EMT), thereby promoting the invasion and metastasis of ESCC. Furthermore, we identified that TTK overexpression can reverse the inhibition of ESCC invasion after ANXA2 knockdown. Overall, these data indicate that the combination of ANXA2 and TTK regulates the activation of the Akt/mTOR pathway and accelerates the progression of ESCC. Therefore, the ANXA2/TTK/Akt/mTOR axis is a potential therapeutic target for ESCC.

## Introduction

Esophageal cancer is an aggressive tumor type that is treated with surgery, chemotherapy, radiotherapy, and other targeted therapies [1]. ESCC is the most common type of esophageal cancer. However, the prognosis of ESCC is still not optimistic. ESCC develops very rapidly. Two-thirds of patients are diagnosed with advanced or metastatic disease, and only one-third of ESCC patients can be surgically resected after initial staging, leading to a diminished quality of life and higher mortality rates among patients [2]. Therefore, it is important to identify molecular markers for early diagnosis and new therapeutic targets for ESCC.

Annexin are calcium- and phospholipid-dependent proteins contributing to many Ca^2+^-dependent membrane-related processes. There are 12 members of the Annexin family (ANXA1–12). ANXA2 is mainly expressed in endothelial cells, monocytes, macrophages, bone marrow cells, and various tumor cells [3], and participates in many vital activities such as trans-membrane transport [4], inflammatory reactions [5, 6], and muscle cell membrane repair [7]. AXNA2 is also closely associated with the development of many cancers [8]. Moreover, abnormal ANXA2 expression in cancer cells has profoundly impacted tumor angiogenesis, tumor cell proliferation, apoptosis, adhesion, invasion, and metastasis [9–12]. Previous studies have shown that ANXA2 is upregulated in many types of cancers and its higher expression levels are associated with poorer overall survival (OS) and shorter disease-free survival (DFS) [13–18]. These results indicate that ANXA2 is a potential diagnostic and prognostic biomarker for various cancers. Furthermore, clinical studies have demonstrated that ANXA2 protein expression is associated with aggressive cancers, drug resistance, and radiotherapy resistance [19], suggesting that ANXA2 may be an oncogene that contributes to the malignant behavior of cancer cells.

TTK, also known as hMps1, is a key mitotic checkpoint protein that segregates chromosomes during mitosis and is essential for cell proliferation and spermatogenesis [20]. TTK is highly expressed during mitosis and in many cancers such as breast [21], lung [22], hepatocellular [23], ovarian cancer [24], and others. Its function in regulating mitosis and its overexpression in many cancers has promoted research on TTK inhibitors as anticancer targets [25]. Our previous study suggested that TTK is overexpressed in hepatocellular carcinoma (HCC) and its expression is closely associated with clinical outcomes [26]. Moreover, TTK plays a role in sorafenib resistance, further suggesting that it could serve as a therapeutic target for HCC [27].

In this report, we provide evidence that the downregulation of ANXA2 in vivo and in vitro can inhibit the progression of ESCC. Results also show that ANXA2 activates the Akt/mTOR signaling pathway and accelerates the EMT process in ESCC through its downstream molecule TTK. Our study is the first to demonstrate interactions between ANXA2 and TTK, as well as their interaction with the Akt/mTOR signaling pathway. In conclusion, our study revealed the mechanism of ANXA2/TTK/Akt/mTOR axis in the progression of ESCC, indicating that ANXA2 and TTK may serve as valuable biomarkers and therapeutic targets for ESCC.

## Materials and methods

### Cell lines

Normal human esophageal epithelial cells (HEEC) and esophageal cancer cells (ECA109, KYSE30, and KYSE520) were cultured in RPIM-1640 medium supplemented (Gibco Laboratories) with 10% fetal bovine serum (FBS) (SERANA, Brandenburg, Germany). HeLa cells were cultured in Dulbecco’s modified eagle medium (DMEM) supplemented with 10% FBS. All cells were purchased from BioeGene (Shanghai, China) and have been kept at the Laboratory of the Clinical Research Center for Cancer of Zhejiang Province. All cell lines were validated using short tandem repeat (STR) profiling and mycoplasma free confirmation.

### siRNA, shRNA, and plasmid transfections

ANXA2 and TTK small interfering RNAs (siRNAs) were purchased from BioeGene (Shanghai, China), FLAG-ANXA2 and MYC-TTK plasmids were purchased from Genomeditech (Shanghai, China), and lentiviral vectors for ANXA2 knockdown were purchased from GeneChem (Shanghai, China). TSnanofect (Tsingke, China) was used to transiently transfect siRNAs and plasmids into esophageal cancer cells and HeLa cells. Esophageal cancer cells were infected with lentivirus by HitransG (GeneChem) to establish stably transfected cell lines. Cells screened for further experiments were continuously cultured at a concentration of 0.5 μg/mL puromycin. The siRNA and shRNA sequences used in this study are listed in Supplementary Table 1.

### Quantitative real-time PCR (qRT-PCR) analysis and primers

Total RNA was isolated using an RNA-Quick Purification Kit (ES Science, China), and a PrimeScript RT Reagent Kit (Accurate Biology, China) was used to reverse transcribe the RNA into cDNA. Quantitative PCR was performed using Accurate Taq Master Mix (Accurate Biology, China) and LightCycler 480 (Roche Diagnostics). Gene expression was calculated based on the 2^−ΔΔCt^ method after standardization with the ACTB gene. All qPCR primers used are listed in Supplementary Table 2.

### Cell proliferation assay

Cells (3 × 10^3^) were seeded into 96-well plates, incubated overnight, and then cultured with Cell Counting Kit-8 (CCK8) reagent (Vazyme, China) at 0, 24, 48, and 72 h according to the manufacturer’s guidelines. Absorbance of 450 nm was detected by a spectrometer (BIOTEK, China) after 2 h.

### Wound healing assay

Cells (3 × 10^5^) were inoculated into 12-well plate, and the middle of each well was scratched with a 10 μL sterile pipette tip. Thereafter, the cells were cultured in RPIM-1640 with 1% FBS. Wound healing distances of 0 and 12 h were determined by ImageJ.

### Cell migration and invasion assays

For migration assays, the transfected cells (5 × 10^4^) were inoculated into Transwell plates (Corning, New York) with 200 μL serum-free medium. Complete culture medium (approximately 700 μL) was added into the lower chamber. Migration cells were fixed with 4% paraformaldehyde after 24 h, stained with 0.1% crystal violet, and counted under a microscope. For invasion assays, the upper chamber was coated with Matrigel (Corning, New York), and then cells (1 × 10^5^) were plated in the upper chamber and cultured in 200 μL serum-free medium with approximately 700 μL complete culture medium was introduced into the lower chamber. After 24 h, migrating cells were fixed, stained, and counted under a microscope.

### Colony formation assay

Cells (1 × 10^3^) were seeded into each well of a 6-well plate and cultured for 2 weeks. Colonies (≥50 cells/colony) were fixed, stained, and counted under a microscope.

### Western blot

Western blot was performed as previously described [28]. Antibodies used were the following: β-actin (1:2000, Cat# GB15003, Servicebio), ANXA2 (1:1000, Cat# F0921, Santa Cruz), TTK (1:1000, Cat# A300-296A, Bethyl), Akt (1:3000, Cat# 4691 T, CST), p-Akt (1:3000, Cat# 4060 T, CST), β-Catenin (1:3000, Cat# 8480 T, CST), Snail (1:3000, Cat# 3879 T, CST), Claudin-1 (1:3000, Cat# 13255 T, CST), mTOR (1:3000, Cat# T55306, Abmart), p-mTOR (1:3000, Cat# T5657, Abmart), Myc-tag (1:3000, Cat# 1:5000, Abmart), and Anti-Flag-tag (1:2000, Cat# A5712, Selleck). An ECL Enhanced Kit (Cat# B520A, Biosharp, China) was used to visualize protein-antibody complexes.

### Co-immunoprecipitation (Co-IP)

Whole-cell protein lysates were collected using cell lysis buffer for Western blot and IP (WBIP) (Cat# P0013, Beyotime, China). For endogenous IP, cell lysates were incubated with Protein A/G Magnetic Beads (Cat# 710078, Selleck, China) and the corresponding antibodies at 4 °C overnight. For exogenous IP, the cell lysates were incubated with Anti-Flag Magnetic Beads (Cat# B26101, Selleck, China) at 4 °C overnight. Immunoprecipitates were washed with WBIP buffer, and then the bound proteins were boiled and analyzed by Western blot.

### Animal tumor models

Shanghai SLAC Experimental Animal Co., Ltd. provided 4-week-old female BALB/c nude mice. Mice were randomly separated into different groups and no blinding method was used for injection. For the tumor metastasis model, KYSE30 cells stably expressing luciferase and infected with sh-ANXA2 or sh-control were injected into mice via the tail vein. For the xenograft assay, ECA109 and KYSE30 (stable ANXA2-knockdown cell lines) with luciferase probes were injected into the right flank of nude mice to establish the proliferation model. Tumor volumes were measured every 2 days for 14 days after tumor formation. Each mouse was injected with 3 mg D-luciferin (Cat# 40902, Yeasen, China), and the tumor status was determined using the IVIS Lumina III imaging system (PerkinElmer). At the end of this period, mice were sacrificed and tumors were excised, weighed, and photographed. All tumors were then embedded in paraffin. Immunohistochemical (IHC) analysis was performed as previously described [29–31], with IHC images scored by pathologists.

### Tissue microarray and IHC staining

The tissue microarray containing ESCC tissue and adjacent normal tissue was purchased from Shanghai Outdo Biotechnology (Shanghai, China). ANXA2 (Cat# F0921, Santa Cruz) and TTK (Cat# T611371, Abmart) were used for IHC staining of the tissue microarray. The staining index is according to the product score of cells staining intensity and the percentage of positive cells (negative=0, weak positive=1, moderate positive=2 and strong positive=3, 0–25% = 1, 26–50% = 2, 51–75% = 3, >75% = 4). Multiply the two scores to obtain the final score result. The product of the two is defined as high expression when it is not less than 6, otherwise, it is defined as low expression.

### Statistical analysis

GraphPad PRISM 8.0 software was used for statistical analyses, with results presented as the mean ± standard deviation (SD). Student’s t-test and one-way or two-way analysis of variance (ANOVA) were performed with a significance level set at *P* < 0.05. Chi-square test was used to analyze the difference of ANXA2/TTK expression levels between ESCC and adjacent normal tissue. The overall survival was assessed by Kaplan-Meier analysis. Pearson’s chi-squared test was used to analyze the correlation between ANXA2 and TTK expression, while a non-parametric test was employed to analyze the relationship between gene expression and clinicopathological variables of ESCC.

## Results

### ANXA2 is upregulated in esophageal cancer and associated with poor clinical outcomes

**Fig. 1:**
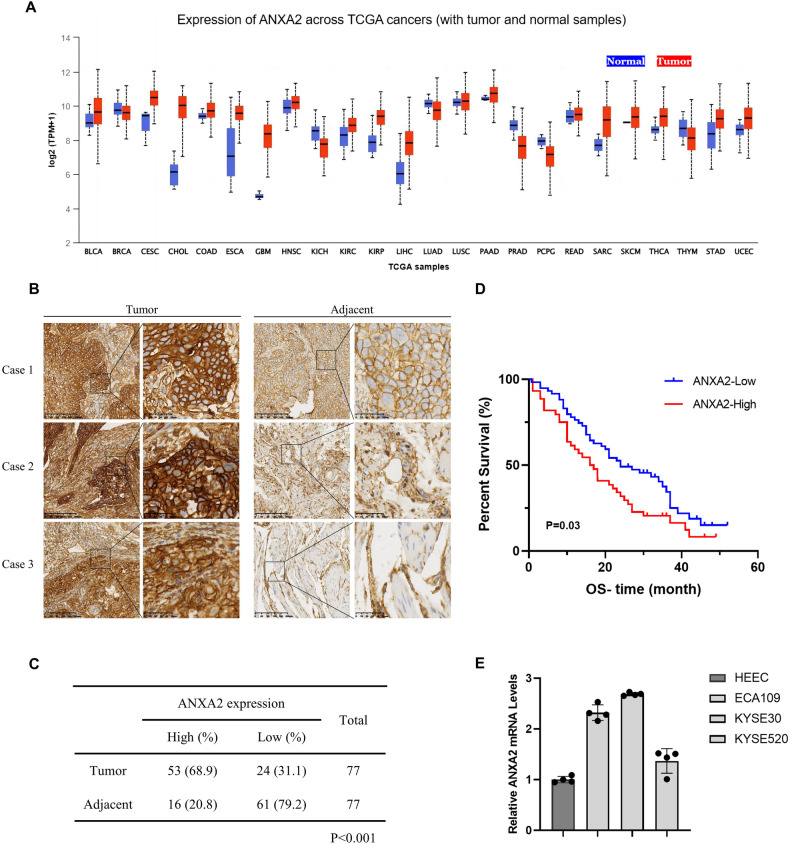
ANXA2 is highly expressed in ESCC and leads to a poor clinical prognosis. **A** The expression difference of ANXA2 in different human cancer types (red columns) and normal tissues (blue columns) in the TCGA database. **B** Representative IHC images of ANXA2 in ESCC tissues. **C** Analysis of IHC results of ANXA2 expression in ESCC. **D** The overall survival showed that high ANXA2 expression was related to the adverse prognosis in ESCC patients. **E** The expression of ANXA2 in HEEC and ESCC cell lines (ECA109, KYSE30, KYSE520) was detected by qRT-PCR.

To investigate abnormal ANXA2 expression in esophageal cancer, we used The UALCAN (http://ualcan.path.uab.edu) and TIMER2.0 (http://timer.comp-genomics.org/) databases to analyze the expression of ANXA2 in tumor and normal tissues (Fig. 1A, Supplementary Fig. 1A). In addition, we performed IHC staining on a tissue microarray containing 77 pairs of ESCC tissues and adjacent normal esophageal tissues. As shown in Fig. 1B, C, ANXA2 is highly expressed in ESCC, and the positive staining rate in ESCC (68.9%) is significantly higher than that in adjacent normal esophageal tissues (20.8%) (*p* < 0.001). These results are consistent with the high expression of ANXA2 in esophageal tumors exhibited in the Human Protein Atlas (https://www.proteinatlas.org/) (Supplementary Fig. 1B). These results suggest that ANXA2 is expressed at higher levels in esophageal cancer than in normal tissues (Supplementary Fig. 1C, D). We further studied the correlation between ANXA2 expression and tumor stage and found that the expression of ANXA2 in tumor stages 1–3 was significantly higher than that in normal tissues (Supplementary Fig. 1E). However, there was no significant correlation between AXNA2 expression and various clinicopathological factors (Supplementary Table 3). Moreover, Kaplan–Meier analysis showed that those with high ANXA2 expression had poorer outcomes compared to patients with low ANXA2 expression (*P* = 0.03) (Fig. 1D). The relationship between ANXA2 expression and clinical outcomes was further verified by GEPIA (http://gepia.cancer-pku.cn/), Kaplan-Meier plotter analysis (https://kmplot.com/analysis/), and OncoLnc databases (http://www.oncolnc.org). Results suggest that higher expression of ANXA2 tends to lead to a worse prognosis (Supplementary Fig. 1F–I). Finally, we determined the mRNA and protein expression levels of ANXA2 in ESCC using qRT-PCR and Western blot. The mRNA and protein levels of ANXA2 in ESCC cell lines (ECA109, KYSE30, and KYSE520) were higher than HEEC (Fig. 1E, Supplementary Fig. 1J). Therefore, our results suggest that ANXA2 acts as an oncogene in ESCC and may serve as a prognostic biomarker in ESCC.

### ANXA2 interacts with TTK and regulates TTK expression

**Fig. 2:**
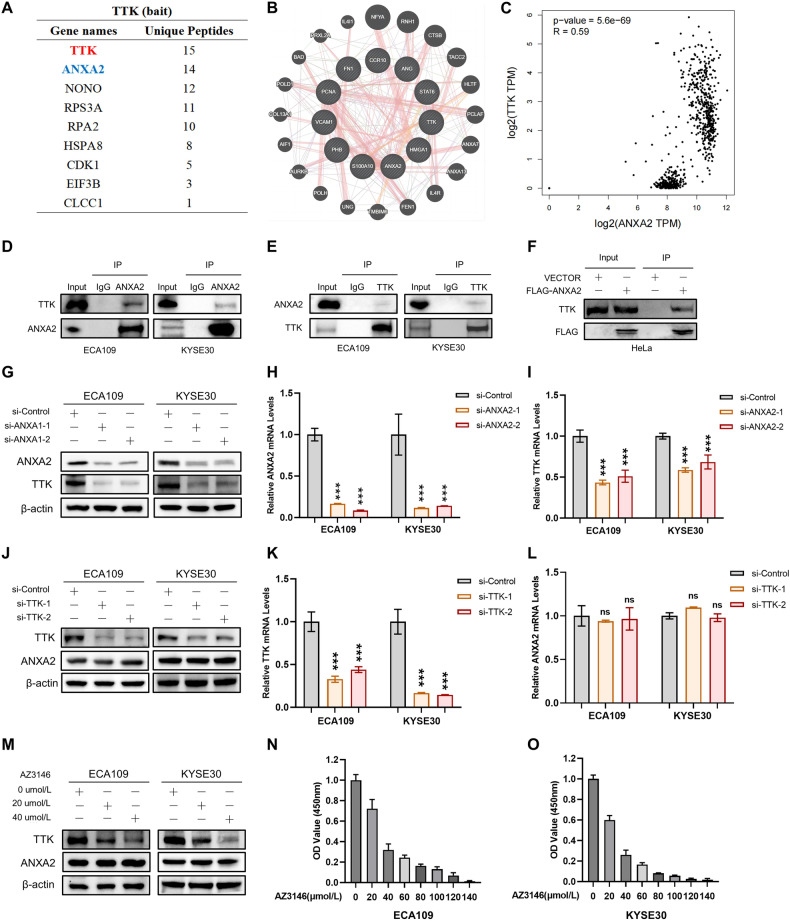
ANXA2 acts as an upstream molecule to regulate TTK protein and mRNA expression. **A** Results of TTK mass spectrometry. **B** Bioinformatics analysis shows that TTK protein interacts with ANXA2 protein. **C** Spearman correlation analysis showed that the expression levels of ANXA2 and TTK were positively correlated. **D**, **E** Co-IP was used to detect the endogenous interaction between ANXA2 and TTK. Rabbit IgG was used as the negative control. **F** Co-IP experiment was carried out with Anti-Flag magnetic beads, and Western blot was carried out with designated antibodies. **G**–**I** Protein and mRNA expression levels of TTK were detected after AXNA2 knockdown. **J**–**L** The protein and mRNA expression levels of ANXA2 were detected after TTK knockdown. **M**–**O** The expression level of ANXA2 was detected after treating ECA109 and KYSE30 cells with AZ3146. *, **, and ***, represent *P* < 0.05, *P* < 0.01, and *P* < 0.001 respectively.

The results of our previous study suggested that the expression of TTK is higher in HCC and is associated with the OS of patients [26]. Here, we investigated proteins that interact with TTK in esophageal cancer by mass spectrometry (Fig. 2A, Supplementary Fig. 2A). In addition, through Genemana (https://genemania.org/) and String (https://string-db.org/) databases, we found that TTK interacts with ANXA2 (Fig. 2B, Supplementary Fig. 2B). Spearman’s correlation analysis revealed a positive correlation between the expression levels of ANXA2 and TTK (Fig. 2C). Co-IP experiment confirmed this interaction. Endogenous interactions between ANXA2 and TTK were observed in the two ESCC cell lines (Fig. 2D–E), and exogenous ANXA2 bound endogenous TTK was observed in the HeLa cells (Fig. 2F). These findings support the results of mass spectrometry analysis and demonstrate that TTK interacts with ANXA2. Next, we explore upstream and downstream relationship between ANXA2 and TTK. We constructed two siRNAs to knockdown endogenous ANXA2 expression. Western blot analysis revealed that the expression of ANXA2 protein was inhibited and leading to a decrease in TTK levels in two distinct ESCC cell lines (Fig. 2G). The change of TTK mRNA level after ANXA2 knockdown was verified by qRT-PCR, and the results were consistent with Western blot. After effectively knocking down the mRNA levels of AXNA2 in the two ESCC cell lines, the mRNA levels of TTK decreased accordingly (Fig. 2H–I). Subsequently, we examined whether TTK regulates ANXA2 protein and mRNA expression. We constructed two siRNA inhibitors targeting endogenous TTK. Results of qRT-PCR showed that we successfully inhibited the mRNA expression level of TTK in ESCC cells. Both Western blot and qRT-PCR results showed that the expression of ANXA2, including protein and mRNA levels remain unchanged (Fig. 2J–L). Furthermore, we explored whether TTK regulates the expression of ANXA2 through AZ3146, a specific inhibitor of TTK [32]. We obtained the IC50 values of the ECA109 and KYSE30 cell lines by CCK8, which were 30.27 μmol/L and 23.88 μmol/L respectively (Fig. 2N, O). TTK protein expression was successfully inhibited by treating the two cell lines with an appropriate drug concentration. However, the expression level of ANXA2 remain unchanged (Fig. 2M). Together, these results indicate that ANXA2 is an upstream regulator of TTK in ESCC.

### TTK overexpression is positively correlated with the up-regulation of ANXA2 in ESCC

**Fig. 3:**
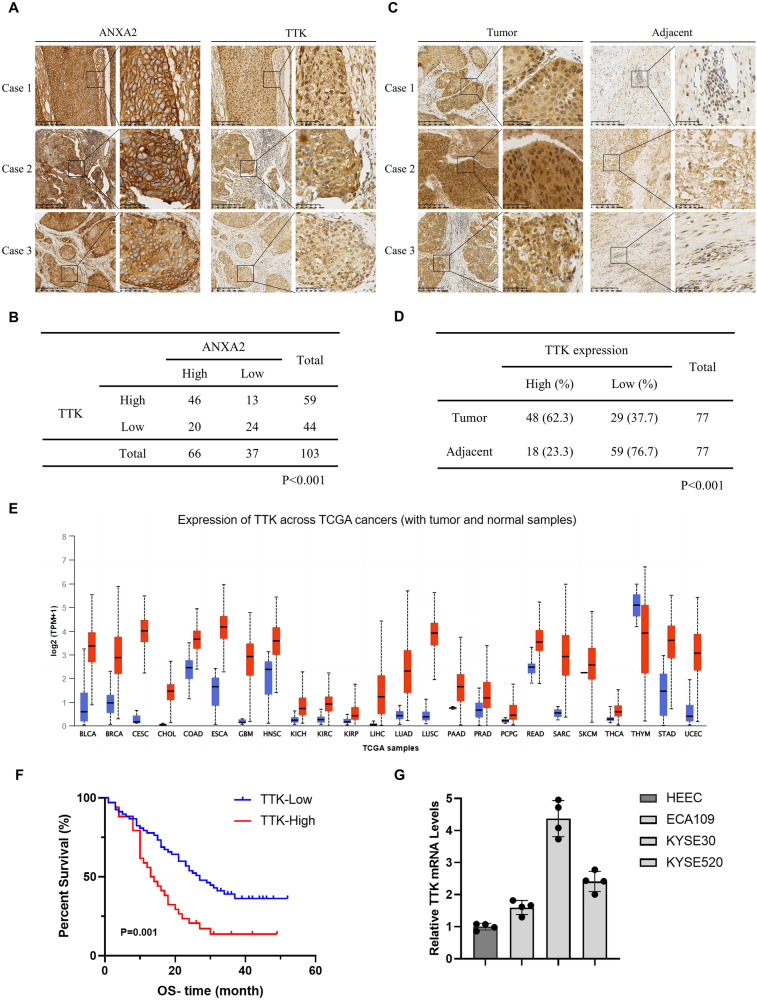
Expressions of TTK in ESCC and its correlation with ANXA2. **A** Representative IHC staining photos of AXNA2 and TTK in ESCC tissue microarray. **B** Statistical analysis indicated that there was a positive correlation between the expression of ANXA2 and TTK in ESCC. **C** Representative photos of IHC staining of TTK in ESCC tissues and adjacent normal tissues. **D** Quantitative analysis of TTK protein level in ESCC tissue microarray. **E** The expression difference of TTK in different human cancer types (red columns) and normal tissues (blue columns) by TCGA database. **F** Overall survival curve showed that high TTK expression was associated with poor prognosis in ESCC patients. **G** The mRNA level of TTK in ESCC cell lines (ECA109, KYSE30, KYSE520) was verified by qRT-PCR.

Furthermore, we performed IHC staining on a tissue microarray containing 77 ESCC tissues and adjacent normal esophageal tissues. By the IHC results of the tissue microarray, a positive correlation between AXNA2 and TTK protein levels was detected (Fig. 3A, B). As indicated in Fig. 3C, D, the expression of TTK in ESCC was higher (62.3%) than in adjacent normal esophagus tissues (23.3%) (*p* < 0.001), which is consistent with the results obtained from the Human Protein Atlas (Supplementary Fig. 3B). UALCAN database and Timer 2.0 were also used to investigate TTK expression in various cancer and normal tissues (Fig. 3E, Supplementary Fig. 3A). The results showed that TTK expression was higher in esophageal cancer tissues than in normal tissues, and TTK expression levels in tumor stages 1–3 were significantly increased (Supplementary Fig. 3C–E). The GEPIA database showed that the DFS and OS of patients with high TTK expression were worse than those of patients with low TTK expression (Supplementary Fig. 3F, G). However, there was no significant correlation between TTK expression and various clinicopathological factors (Supplementary Table 4). Furthermore, the results from ESCC tissue microarray and PrognoScan database (http://dna00.bio.kyutech.ac.jp/PrognoScan/) show that the up-regulation of TTK leads to a lower survival rate of ESCC patients (Fig. 3F, Supplementary Fig. 3H). Further, qRT-PCR results also confirmed that the TTK mRNA level was upregulated in ESCC cell lines (Fig. 3G). These results indicate that TTK is a carcinogenic gene with the potential to predict ESCC prognosis.

### ANXA2 promotes the malignant biological function of ESCC in vitro

**Fig. 4:**
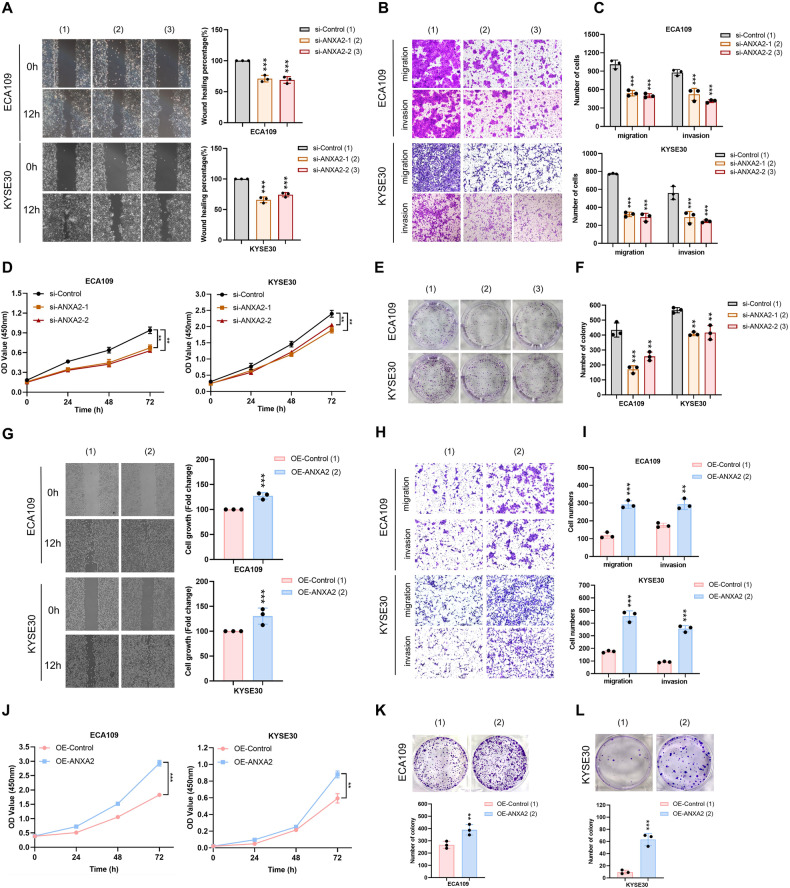
ANXA2 regulates the malignant biological behavior of esophageal cancer cells. **A** Wound healing assay was used to evaluate the migration in the ANXA2 knockdown group and control group. **B**, **C** Transwell assay was employed to study the migration and invasion ability of two kinds of ESCC cells after ANXA2 knockdown. **D** The cell viability of the control group and si-ANXA2 group was determined by CCK8 assay. **E**, **F** The colony formation assay was carried out to clarify the proliferation ability of ESCC cells in the control group and AXNA2 knockdown group. **G** Wound healing assay was employed to testify the migration ability of ANXA2 overexpressed cells. **H**, **I** Changes of cell migration and invasion ability after ANXA2 overexpression were detected by transwell assay. **J** CCK8 assay was used to detect the cell viability after ANXA2 overexpression. **K**, **L** Colony formation was used to evaluate the effect of ANXA2 overexpression on cell proliferation. *, **, and ***, represent *P* < 0.05, *P* < 0.01, and *P* < 0.001, respectively.

To verify the role of ANXA2 in regulating the progression of ESCC, we inhibited endogenous ANXA2 expression in ECA109 and KYSE30 cell lines. The effect of ANXA2 on the migration and invasion of ESCC cells was assessed by wound healing and transwell assays. A wider wound healing area and fewer migrating cells were observed in the ANXA2-knockdown group (Fig. 4A–C). Second, colony formation and CCK8 assays were employed to evaluate the proliferative ability of ESCC cells. We found that the cell viability and proliferation in the ANXA2-knockdown group were lower than in the control group (Fig. 4D–F). Furthermore, we transfected ESCC cells with an ANXA2 overexpression plasmid. Wound healing and transwell assays showed that the migration and invasion abilities of ANXA2 overexpression cancer cells were enhanced (Fig. 4G–I). Results of CCK8 and colony formation assays indicated that cell viability and proliferation ability were higher in the ANXA2 overexpression group compared to the control group (Fig. 4J–L). As a result, ANXA2 can regulate the proliferation, invasion, and metastasis of ESCC cell lines, suggesting that ANXA2 may be involved in the development of ESCC.

### ANXA2 and TTK can both activate Akt/mTOR signaling and regulate EMT-related proteins

**Fig. 5:**
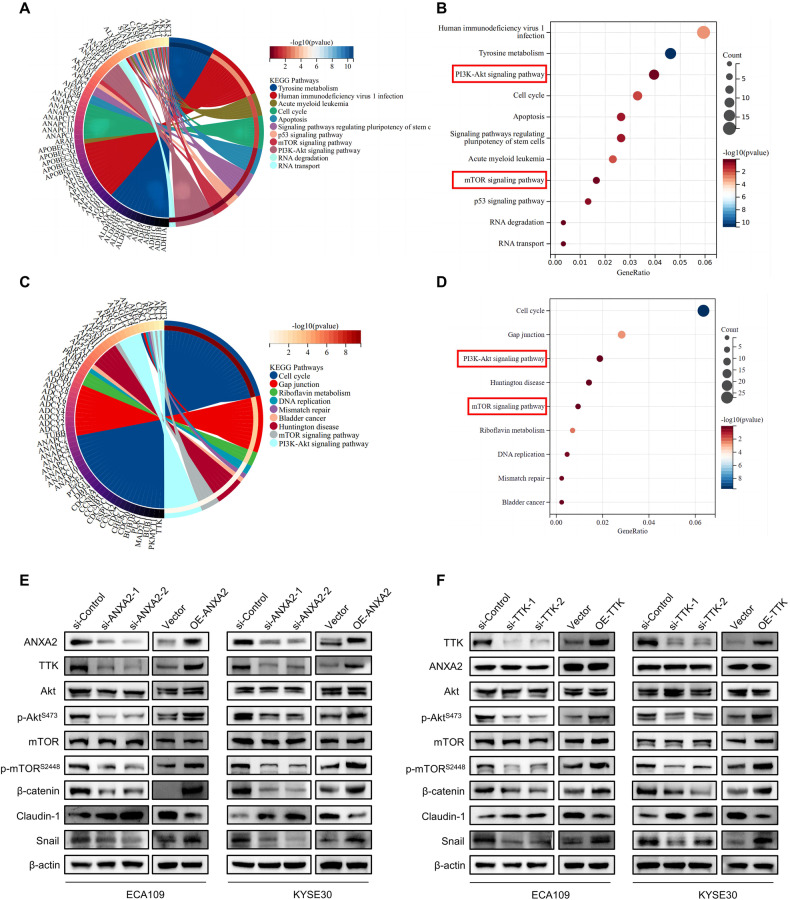
ANXA2 and TTK activate the Akt/mTOR signaling pathway and EMT process. **A**–**D** The GO annotation of genes in the R software package orq.Hs.eq.db (version 3.1.0) and clusterProfiler (version 3.14.3) were used for potential function and pathway analysis. **E** The changes of downstream proteins after ANXA2 knockdown and overexpression were detected by Western blot. **F** Western blot results showed that TTK regulates the expression of EMT-related proteins and Akt/mTOR signaling pathway in ESCC.

The mechanism of ANXA2 in regulating the biological behavior of ESCC was further studied. We obtained potential pathways and targets of ANXA2 through Gene Ontology (GO) and Kyoto Encyclopedia of Genes and Genomes (KEGG) analyses. As a result, ANXA2 was found to regulate the PI3K/Akt and mTOR pathways (Fig. 5A, B). GO and KEGG were also used to explore the potential signaling pathways regulated by TTK. The results showed that TTK could also regulate the PI3K/Akt and mTOR pathways (Fig. 5C, D). Subsequently, endogenous ANXA2 was inhibited and overexpressed in ECA109 and KYSE30 cell lines, and associated protein changes were detected by Western blot. When ANXA2 was effectively knocked down, we found that levels of phosphorylated Akt (p-Akt) and phosphorylated mTOR (p-mTOR) decreased, whereas total Akt and total mTOR levels remained constant. In addition, the expression levels of EMT-related proteins such as β-catenin and Snail decreased, while the protein level of Claudin-1 increased (Fig. 5E). We verified the reliability of this pathway by overexpressing exogenous ANXA2. The protein levels of p-Akt and p-mTOR increased after ANXA2 overexpression, while the total Akt and total mTOR levels remained unchanged. Moreover, the protein levels of β-catenin and Snail increased and Claudin-1 decreased (Fig. 5E). ECA109 and KYSE30 cells were also transfected with TTK-targeted siRNAs and their corresponding overexpression plasmids. Western blot was used to detect relevant changes in pathway and EMT-related proteins. When TTK was inhibited or overexpressed in ECA109 and KYSE30 cells, the levels of p-Akt and p-mTOR changed, while the levels of total Akt and total mTOR remained constant. The protein expression levels of β-catenin, Snail, and Claudin-1 were also measured. β-catenin and Snail protein levels were decreased in the TTK knockdown group, whereas Claudin-1 protein levels increased (Fig. 5F), this result suggests that TTK may also activate Akt/mTOR signaling pathway and regulate the EMT process. These findings indicate that ANXA2 and TTK can accelerate ESCC progression by activating the Akt/mTOR signaling pathway and regulating EMT-related proteins.

### TTK is involved in ESCC progression

**Fig. 6:**
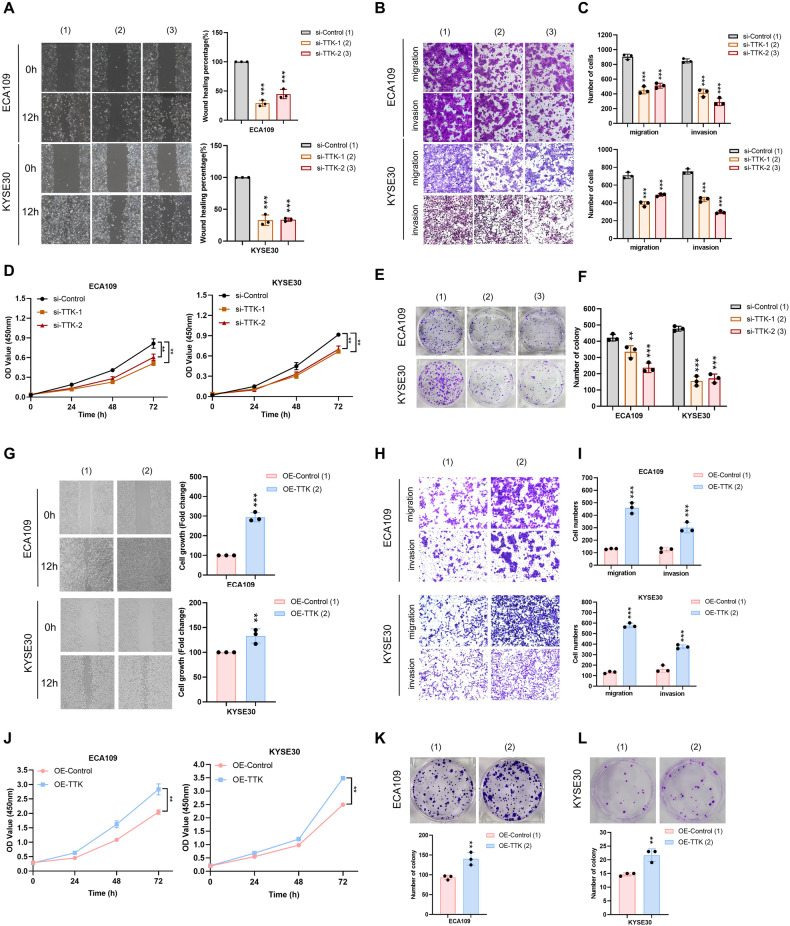
TTK regulates ESCC proliferation, migration and invasion. **A**–**C** Wound healing and transwell assay were used to verify the difference of migration and invasion ability between the TTK knockdown group and the control group. **D** The change of cell viability after TTK knockdown was demonstrated by CCK8 assay. **E**, **F** Colony formation was used to measure the effect of TTK on cell proliferation. **G** The migration ability of cells in the control group and TTK overexpression group was proved by wound healing assay. **H**, **I** The ability of cell migration and invasion in the control group and TTK overexpression group was evaluated by transwell assay. **J** CCK8 assay was used to detect the cell viability of the TTK overexpression group and control group. **K**, **L** Cell proliferation ability was measured by colony formation assay. *, **, and ***, represent *P* < 0.05, *P* < 0.01, and *P* < 0.001 respectively.

The potential role of TTK in the malignant progression of ESCC was further studied by wound healing and transwell assays. Results showed that inhibiting the expression of endogenous TTK reduced the migration and invasion abilities of ECA109 and KYSE30 cells (Fig. 6A–C). CCK8 and colony formation assays indicated that the proliferation ability of ESCC cells in the TTK knockdown group was lower than that in the control group (Fig. 6D–F). Subsequently, we used CCK8, wound healing, transwell, and colony formation assays to assess alterations in the biological behavior of ESCC cells after TTK overexpression. It was found that the migration and invasion abilities of ESCC cells in the TTK overexpression group were higher than control group (Fig. 6G–I). In addition, cell proliferation ability was also increased in the TTK overexpression group (Fig. 6J–L). These results suggest that TTK promotes the malignant behavior of ESCC cells.

### TTK is required in ANXA2-mediated ESCC progression

**Fig. 7:**
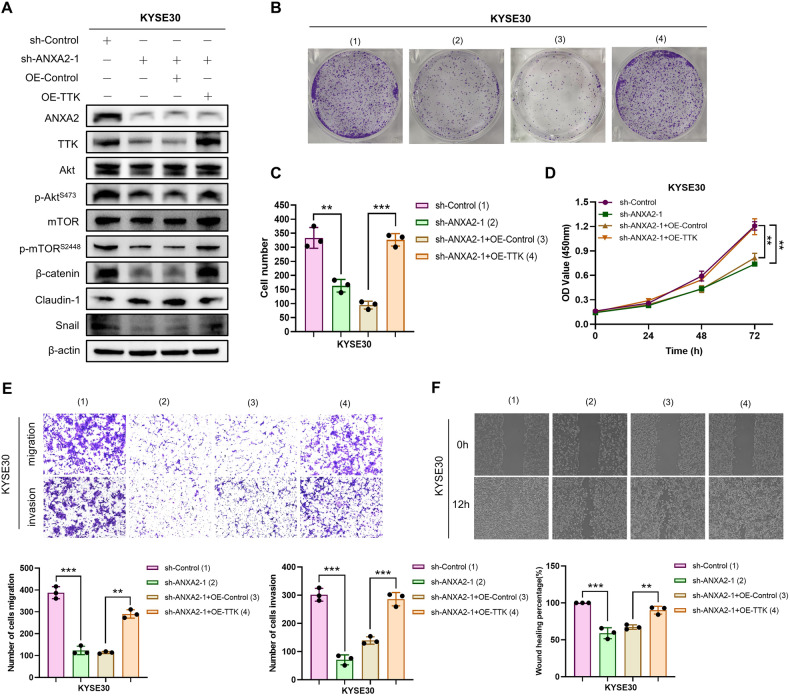
AXNA2 partially relies on TTK to promote ESCC development. **A** Protein changes in different treatment groups were detected by Western blot. **B**–**D** The colony formation and CCK8 assay were used to detect the rescue effect of TTK overexpression on ANXA2 deficiency. (**E**, **F**) Wound healing and transwell assay explored the migration and invasion ability of ESCC in different treatment groups. *, **, and ***, represent *P* < 0.05, *P* < 0.01, and *P* < 0.001, respectively.

Previous studies have indicated that ANXA2 and TTK may play important roles in ESCC progression, with TTK being identified as the downstream target of ANXA2. We aimed to determine whether TTK is essential for ANXA2 to exert its function in ESCC. We transfected the TTK overexpression and control plasmids into KYSE30 cells (stable ANXA2-knockdown cell line). Western blot results of p-Akt and p-mTOR showed that TTK overexpression rescued the effects of ANXA2 deficiency on the Akt/mTOR pathway (Fig. 7A). Furthermore, the inhibitory effect of ANXA2 down-regulation on EMT was completely restored by TTK up-regulation (Fig. 7A). The results of colony formation and CCK8 assays indicated that upregulating TTK increased the proliferation ability of ESCC cells, which had been inhibited by ANXA2 knockdown (Fig. 7B–D). In addition, wound healing and transwell assays revealed that TTK up-regulation rescued the suppressive effects of ANXA2 knockdown on cell migration and invasion (Fig. 7E, F). In conclusion, ANXA2 promotes proliferation, migration, invasion, Akt/mTOR signaling pathway, and EMT process by regulating TTK expression in ESCC.

### ANXA2 promotes the progression of ESCC in vivo

**Fig. 8:**
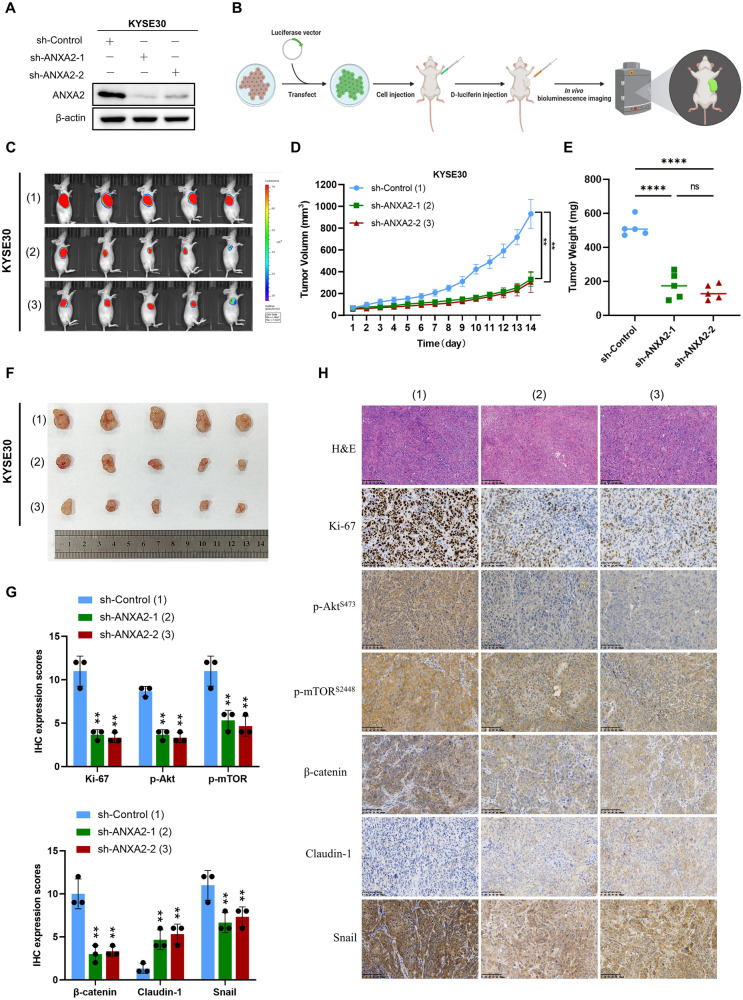
Suppression of ANXA2 inhibited tumor growth in vivo. **A** Western blot analysis showed that ANXA2 was effectively knocked down in KYSE30. **B** Schematic diagram of in vivo tumor model construction. **C** Bioluminescence images of mice after subcutaneous injection of KYSE30 transfected cells after 28 days. **D** Growth curves of three groups of xenograft tumors (tumor volume were calculated by the following formula: V = W^2^*L/2). **E** Analysis of tumor weight of three groups of xenograft tumors. **F** After 28 days, the mice were sacrificed and the tumor photographs were taken. **G**, **H** IHC analysis was employed to evaluate the expression of Ki-67, p-Akt, p-mTOR, β-catenin, Claudin-1 and Snail, and H&E staining was used to grade malignant tumors. *, **, and ***, represent *P* < 0.05, *P* < 0.01, and *P* < 0.001, respectively.

To study the clinical effects of ANXA2 in ESCC, we use shRNAs to establish stable ANXA2-knockdown cancer cells transfected with a luciferase reporter gene plasmid. As shown in Fig. 8A, we successfully suppressed AXNA2 protein expression in KYSE30 cell line. KYSE30 cells with stable ANXA2 knockdown were injected into the subcutaneous area of the armpits of nude mice, tumor sizes were observed using the IVIS Lumina III imaging system (Fig. 8B). Bioluminescence images showed that the tumor size in the ANXA2 knockdown group was smaller than that in the control group (Fig. 8C). Tumor growth curve results showed that ANXA2 knockdown inhibited the size and growth rate of tumors in vivo (Fig. 8D). At the end of observation, mice were sacrificed and subcutaneous tumors were excised, weighed, and photographed (Fig. 8E, F). Subsequently, KYSE30 cells stably transfected with sh-ANXA2 or sh-Control were injected into the nude mice via the tail vein. After 30 days, bioluminescence imaging was performed using the IVIS Lumina III imaging system. The results showed that ANXA2 knockdown significantly reduced the distant metastasis ability of ESCC (Supplementary Fig. 4A, B). Furthermore, ECA109 stable ANXA2-knockdown cancer cells were subcutaneously injected into the right flank of nude mice. The results of tumor volume and weight analysis showed that the tumor in the ANXA2 knockdown group was smaller than the control group (Supplementary Fig. 4C, D). Sections of sh-control and sh-AXNA2 xenograft tumors were stained with hematoxylin and eosin (H&E), and tumorigenic markers were detected by IHC. Expression of Ki-67, a key proliferation marker, was also higher in the control group. The IHC results showed that the staining intensities of p-Akt and p-mTOR in the sh-AXNA2 groups were lower than the control group (Fig. 8G, H). Besides, the results of IHC showed that the staining intensity of β-catenin and Snail in the ANXA2-knockdown group was lower compared to the control group. However, the staining intensity of Claudin-1 was higher than that in the control group (Fig. 8G, H). All these dates reveal that ANXA2-konckdown reduced the expression of p-Akt, p-mTOR, β-catenin and Snail while increasing the expression of Claudin-1. In conclusion, AXNA2 promotes the growth and proliferation of ESCC by up-regulating Ki-67 and activating the Akt/mTOR signaling pathway.

## Discussion

Several evidences show that ANXA2 plays a role as a carcinogen in ESCC. First, ANXA2 is overexpressed in ESCC, and higher ANXA2 expression level is associated with worse clinical prognosis. Secondly, ANXA2 promotes the growth and metastasis of ESCC in vivo and in vitro. Third, ANXA2 promotes the progression of ESCC through TTK and a series of related factors, including the Akt/mTOR signaling pathway and EMT-related proteins such as β-catenin, Snail and Claudin-1.

Recent studies have revealed the role of ANXA2 in various tumors. ANXA2 is regulated by CircADAMTS6, S100A11, and miR155HG in glioblastoma (GBM) and promotes the progression of GBM [33–35]. Meanwhile, various types of non-coding RNA have been discovered to regulate ANXA2 expression, including LINC00659, LINC01133, lncRNA-MUF, and MIR155HG [36–39]. In HCC and breast cancer, ANXA2 was found to interact with FBXW7 and MIEN2 respectively [40, 41]. Besides, ANXA2 can participate in the formation of various compounds to promote drug resistance in breast cancer [42–44]. ANXA2 is also associated with several classical cancer-related pathways such as PI3K/Akt signaling, Akt/mTOR signaling, and NF-κB signaling pathways in lung cancer, gastric cancer, and GBM respectively [34, 45, 46]. Regrettably, there have been few studies on ANXA2 in esophageal cancer. According to Ma et al., ANXA2 and its activation of the MYC-HIF1A-VEGF signaling pathway are important factors in promoting the metastasis of esophageal cancer [47]. Furthermore, the loss of E3 ubiquitin ligase FBXW7 function has also been shown to lead to an increase in ANXA2 expression levels, thus promoting the malignant progression of esophageal cancer through ERK activation [48]. These results confirm that ANXA2 plays an essential role as an oncogene in esophageal cancer. However, further studies on the regulatory mechanisms of ANXA2 and specific biological processes involved in esophageal cancer are still required. For the first time, we have discovered that the ability of AXNA2 to enhance ESCC cell proliferation both in vivo and in vitro is associated with the activation of the Akt/mTOR signaling pathway. Our results indicate that ANXA2 activates the Akt/mTOR signaling pathway and EMT in ESCC, thereby enhancing cancer invasion and metastasis through the regulation of β-catenin, Snail, and Claudin-1 proteins.

In our study, we first reported that ANXA2 interacts with TTK and acts as a molecule upstream of TTK, regulating ESCC cell growth and metastasis. Chen et al. reported that TTK inhibits apoptosis through the Akt/mTOR pathway in ovarian cancer [49]. Besides, TTK was found to regulate tumor proliferation in gastric cancer, colon cancer and HCC [50–52]. TTK has also been reported as a downstream molecule of microRNA-335-5p in esophageal cancer [53]. The potential mechanism of action of TTK in esophageal cancer, however, has not been thoroughly studied. Our results showed that TTK can promote the proliferation and metastasis of ESCC by regulating β-catenin, Snail, and Claudin-1. Furthermore, TTK is involved in the activation of the Akt/mTOR signaling pathway in ESCC. It is worth noting that TTK can rescue the decline in metastatic ability of ESCC caused by ANXA2 inhibition, indicating that TTK plays an indispensable role in the malignant biological function of ANXA2. Further research is required to identify the molecular mechanisms underlying the interaction between ANXA2 and TTK in other cancers.

ANXA2 has been identified as a potential prognostic biomarker in many cancers such as breast cancer and HCC [54–57]. Co-expression of ANXA2 with HOXA13 and SOD2 has also been found to play a prognostic role in esophageal cancer [58]. Our findings indicate that ANXA2 regulates the expression of EMT-related proteins in ESCC. EMT is the reason cancer cells evade drug treatment and continue to metastasis [59]. Therefore, therapeutic methods aimed at inhibiting EMT have great potential for preventing cancer metastasis and treating drug resistance. Currently, a small molecular function inhibitor of ANXA2, 5α-epoxyalantolactone (5α-EAL) is expected to become an effective treatment for breast cancer [60]. Therefore, we suspect that ANXA2 has the potential to be an EMT inhibitor and a new therapeutic target in ESCC. The ability of TTK to promote mitotic stability can be exploited for cancer treatment strategies [23]. Nowadays, five small molecule inhibitors of TTK are currently under clinical study for head and neck, endocrine, gastrointestinal, genitourinary, gynecologic, and breast cancers, as well as melanomas and sarcomas of the soft tissue and bone [61–65].

## Conclusion

**Fig. 9:**
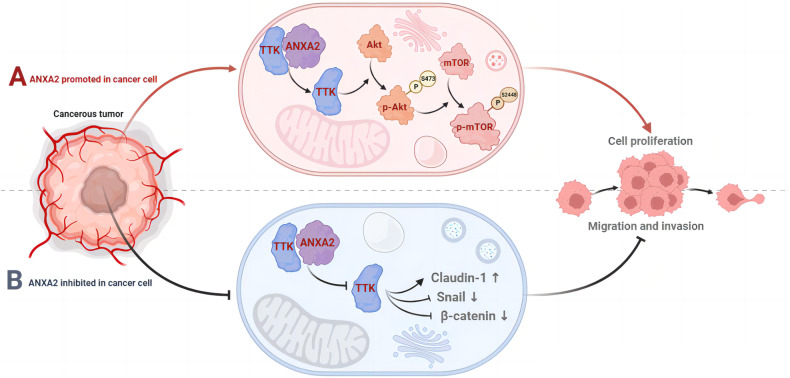
The molecular mechanism of ANXA2 in ESCC.

In summary, our work found that ANXA2 acts as an upstream molecule and plays an indispensable role in regulating TTK, which provides a new perspective for understanding the molecular mechanism of ANXA2 and the biological significance of the Akt/mTOR signaling pathway mediated by ANXA2 and TTK in ESCC (Fig. 9). More importantly, we also provided evidence that ANXA2 plays a carcinogenic role in ESCC by downstream target TTK. As the expression of ANXA2 and TTK is up-regulated in clinical tumor specimens, and they are both closely related to high tumor stage and poor prognosis, targeting ANXA2 and TTK may be new therapeutic strategies for ESCC. In the future, it is necessary to explore the potential of targeted drug therapy strategies based on AXNA2 and TTK in ESCC.

### Supplementary information

Supplementary Figure 1

Supplementary Figure 2

Supplementary Figure 3

Supplementary Figure 4

Supplementary Figure 5

Supplementary tables

Original data file

## Data Availability

The data that support the findings of this study are available from the corresponding author upon reasonable request.
