# Supplementary figures and images for: Annexin A2 combined with TTK accelerates esophageal cancer progression via the Akt/mTOR signaling pathway
Source: Cell Death Dis. 2024 Apr 24;15(4):291. doi: 10.1038/s41419-024-06683-w (PMC11043348; doi:10.1038/s41419-024-06683-w)

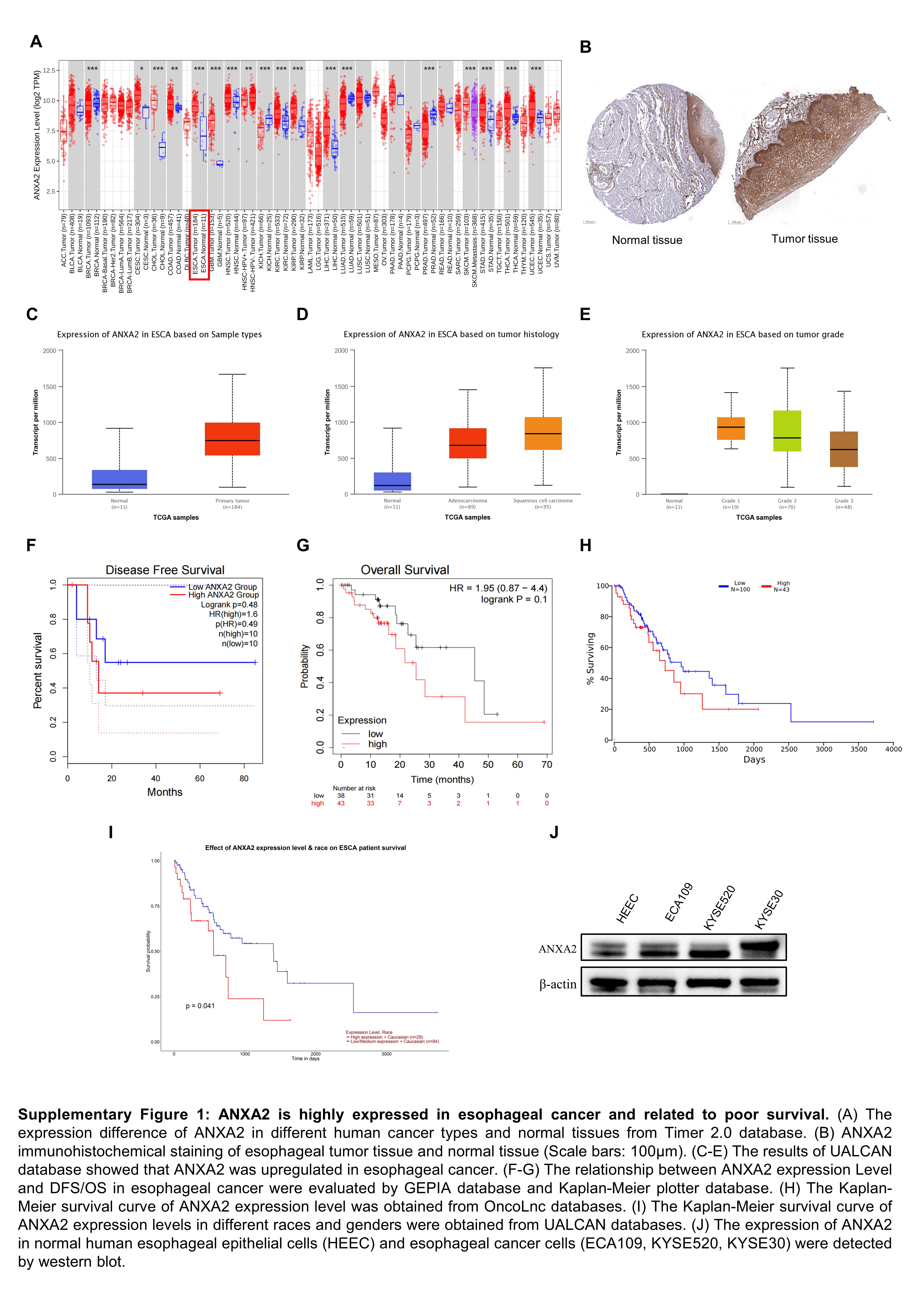

Supplement: Supplementary file 1 — Supplementary Figure 1 [file 41419_2024_6683_MOESM1_ESM.jpg]

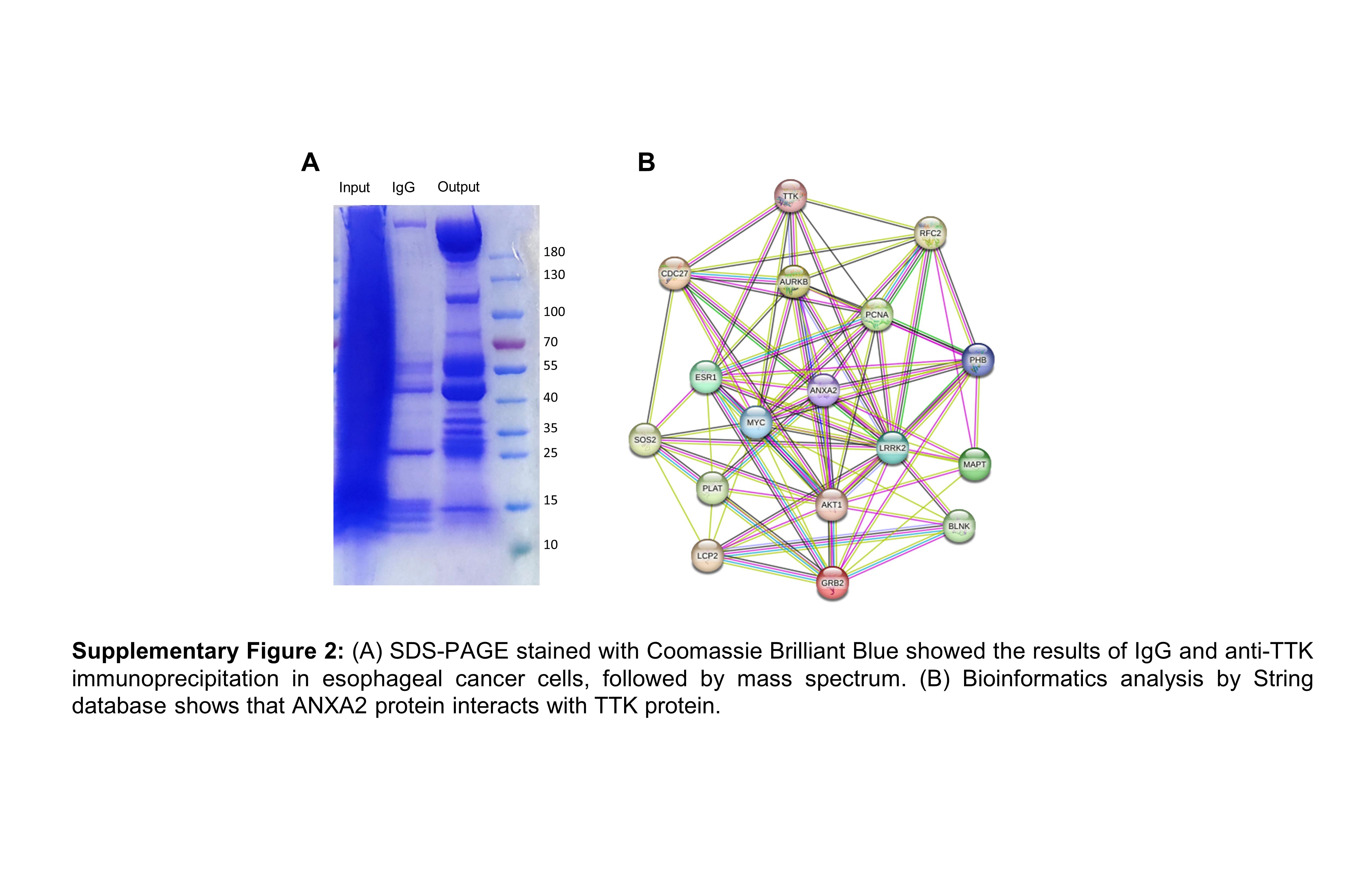

Supplement: Supplementary file 2 — Supplementary Figure 2 [file 41419_2024_6683_MOESM2_ESM.jpg]

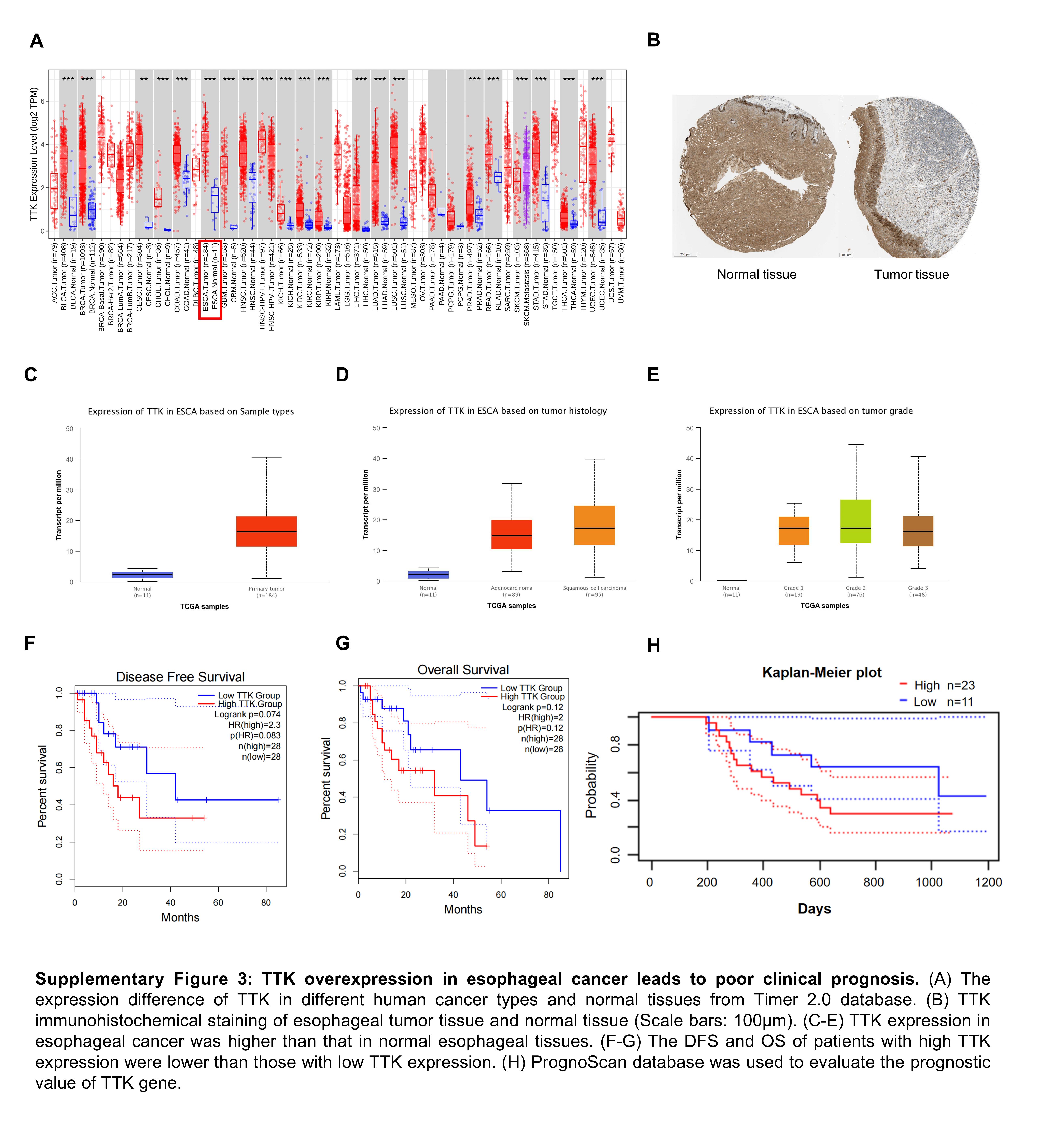

Supplement: Supplementary file 3 — Supplementary Figure 3 [file 41419_2024_6683_MOESM3_ESM.jpg]

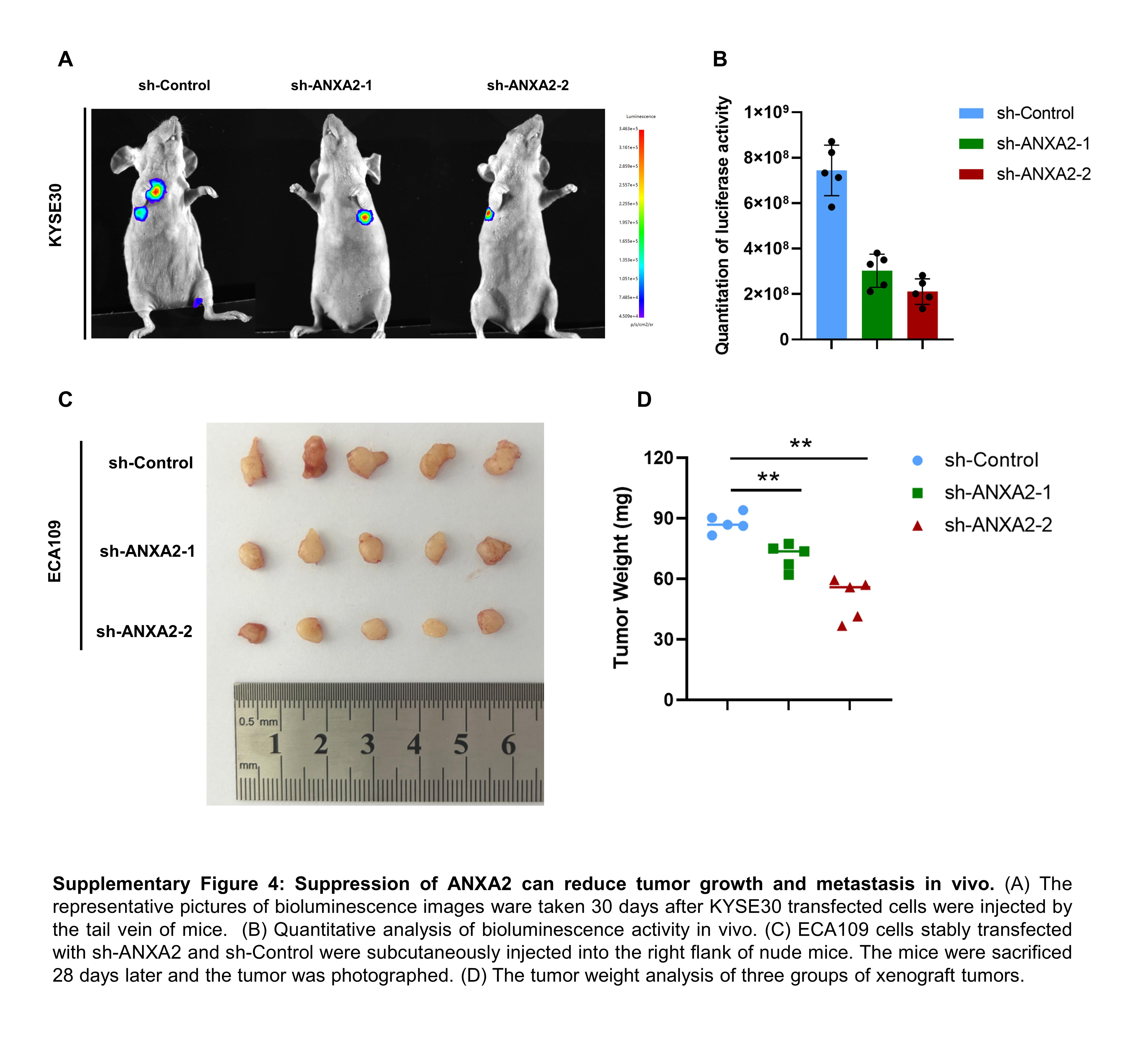

Supplement: Supplementary file 4 — Supplementary Figure 4 [file 41419_2024_6683_MOESM4_ESM.jpg]

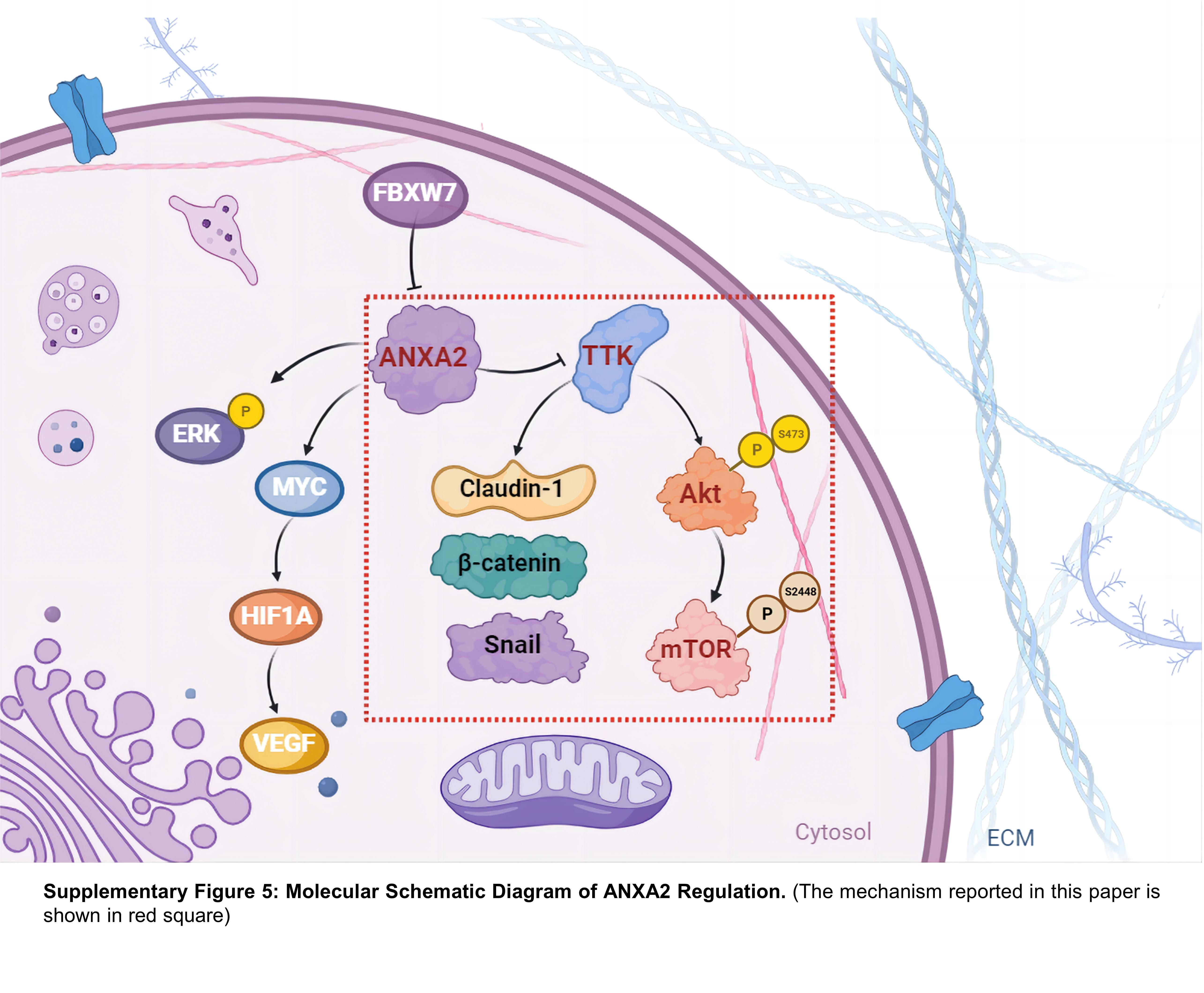

Supplement: Supplementary file 5 — Supplementary Figure 5 [file 41419_2024_6683_MOESM5_ESM.jpg]
