# Supplementary material for: Annexin A2 combined with TTK accelerates esophageal cancer progression via the Akt/mTOR signaling pathway
Source: Cell Death Dis. 2024 Apr 24;15(4):291. doi: 10.1038/s41419-024-06683-w (PMC11043348; doi:10.1038/s41419-024-06683-w)
Supplement: Supplementary file 6 — Supplementary tables [file 41419_2024_6683_MOESM6_ESM.docx]

**Supplementary Table 1: siRNA and shRNA sequences**

| Targeted Genes | Sequences |
| --- | --- |
| si-TTK-NC | TTCTCCGAACGTGTCACGT |
| si-TTK-1 | GCAATACCTTGGATGATTA |
| si-TTK-2 | GCACGTGACTACTTTCAAA |
| si-ANXA2-NC | TTCTCCGAACGTGTCACGT |
| si-ANXA2-1 | CGGCTGTATGACTCCATGA |
| si-ANXA2-2 | GACCAACCGCAGCAATGCA |
| sh-ANXA2-NC | TTCTCCGAACGTGTCACGT |
| sh-ANXA2-1 | TGTGGATGAGGTCACCATTGT |
| sh-ANXA2-2 | GAAAGCATCAGGAAAGAGGTT |

**Supplementary Table 2: qPCR primers sequences**

| Primer name | Primer sequence (5' to 3') |
| --- | --- |
| ACTB F | CATGTACGTTGCTATCCAGGC |
| ACTB R | CTCCTTAATGTCACGCACGAT |
| ANXA2 F | GAGCGGGATGCTTTGAACATT |
| ANXA2 R | TAGGCGAAGGCAATATCCTGT |
| TTK F | GTGGAGCAGTACCACTAGAAATG |
| TTK R | CCCAAGTGAACCGGAAAATGA |

**Supplementary Table 3: Correlation between clinicopathological factors and ANXA2 expression in ESCC patients**

| **Features** | **ANXA2 low expression** | **ANXA2 high expression** | **P-value** |
| --- | --- | --- | --- |
| **Sex** |  |  |  |
| **Male** | **28** | **57** | **0.173** |
| **female** | **9** | **9** |  |
| **Stage** |  |  |  |
| **Ⅰ-Ⅱ** | **34** | **51** | **0.062** |
| **Ⅲ-Ⅳ** | **3** | **15** |  |
| **T** |  |  |  |
| **1** | **0** | **1** | **0.852** |
| **2** | **5** | **8** |  |
| **3** | **30** | **52** |  |
| **4** | **2** | **5** |  |
| **N** |  |  |  |
| **0** | **14** | **27** | **0.607** |
| **1** | **17** | **19** |  |
| **2** | **4** | **15** |  |
| **3** | **2** | **5** |  |
| **M** |  |  |  |
| **0** | **37** | **63** | **0.190** |
| **1** | **0** | **3** |  |

**Supplementary Table 4: Correlation between clinicopathological factors and TTK expression in ESCC patients**

| **Features** | **TTK low expression** | **TTK high expression** | **P-value** |
| --- | --- | --- | --- |
| **Sex** |  |  |  |
| **Male** | **38** | **44** | **0.316** |
| **female** | **6** | **12** |  |
| **Stage** |  |  |  |
| **Ⅰ-Ⅱ** | **39** | **42** | **0.017** |
| **Ⅲ-Ⅳ** | **4** | **17** |  |
| **T** |  |  |  |
| **1** | **0** | **1** | **0.184** |
| **2** | **9** | **4** |  |
| **3** | **32** | **50** |  |
| **4** | **3** | **4** |  |
| **N** |  |  |  |
| **0** | **18** | **23** | **0.265** |
| **1** | **19** | **17** |  |
| **2** | **6** | **13** |  |
| **3** | **1** | **6** |  |
| **M** |  |  |  |
| **0** | **43** | **57** | **0.740** |
| **1** | **1** | **2** |  |
