# Supplementary material for: Annexin A2 combined with TTK accelerates esophageal cancer progression via the Akt/mTOR signaling pathway
Source: Cell Death Dis. 2024 Apr 24;15(4):291. doi: 10.1038/s41419-024-06683-w (PMC11043348; doi:10.1038/s41419-024-06683-w)
Supplement: Supplementary file 7 — Original data file [file 41419_2024_6683_MOESM7_ESM.pdf]

Figure 2D

TTK

ANXA2

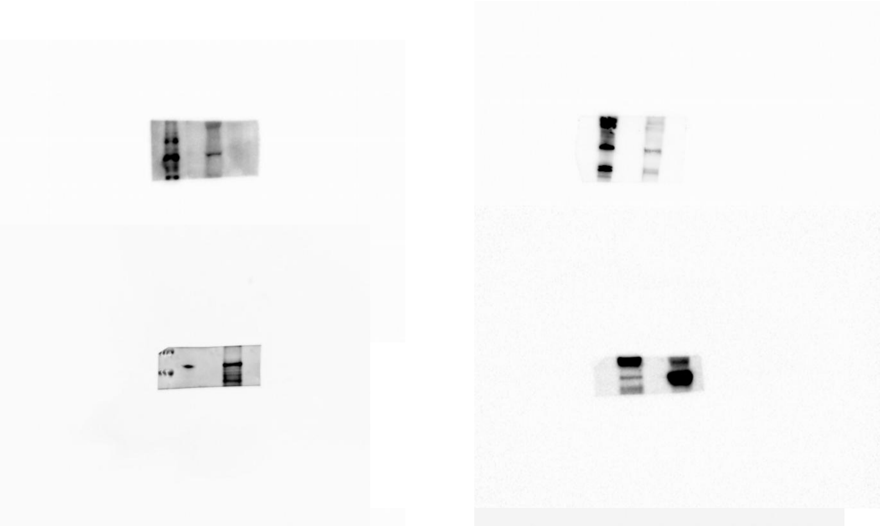

Figure 2E

TTK

ANXA2

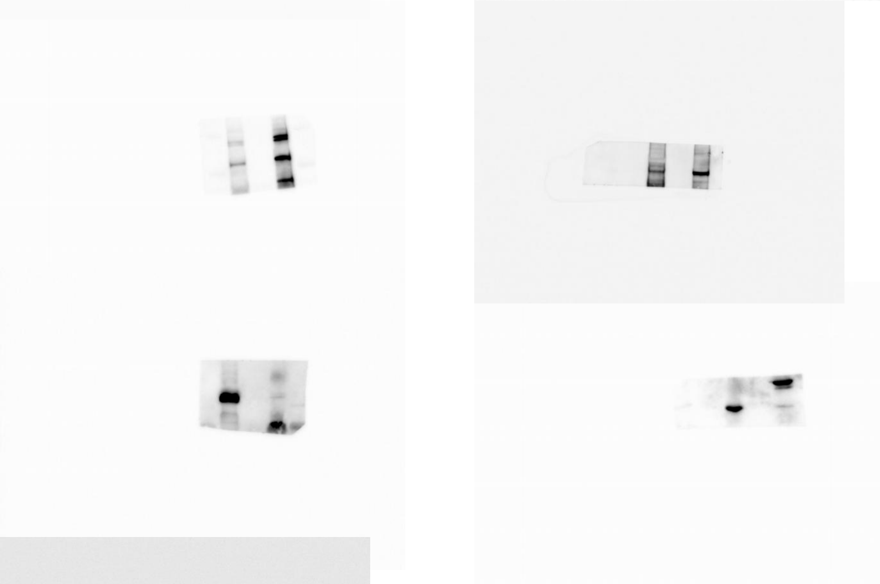

Figure 2F

TTK

FLAG

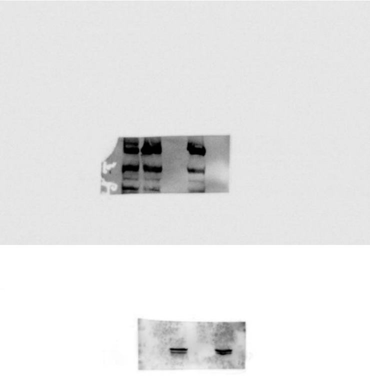

Figure 2G

ANXA2

TTK

β-actin

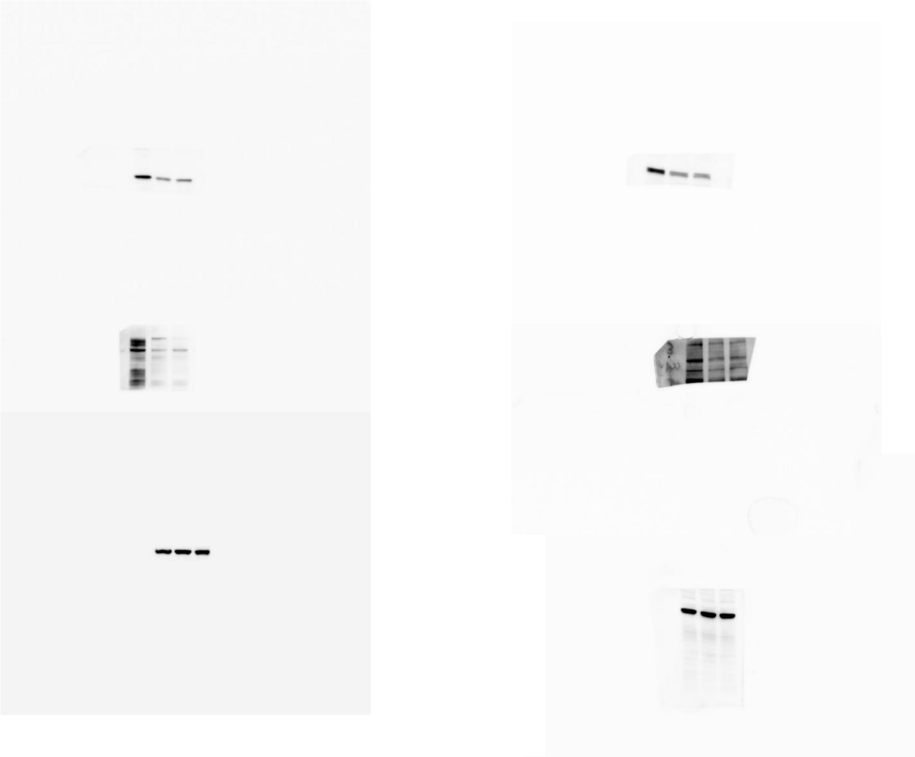

Figure 2J

ANXA2

TTK

β-actin

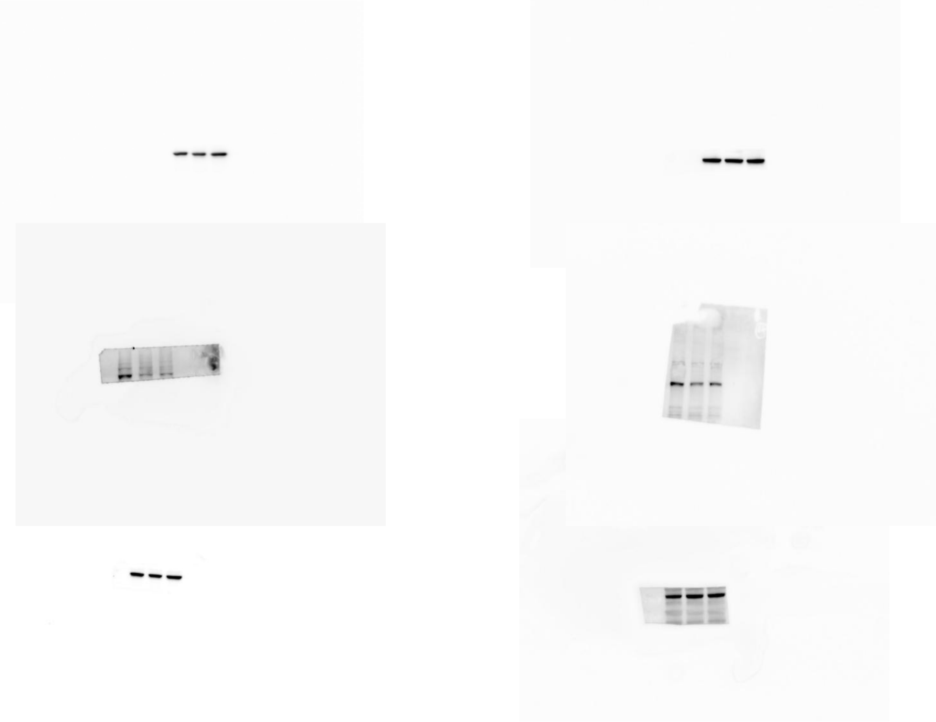

Figure 2M

ANXA2

TTK

β-actin

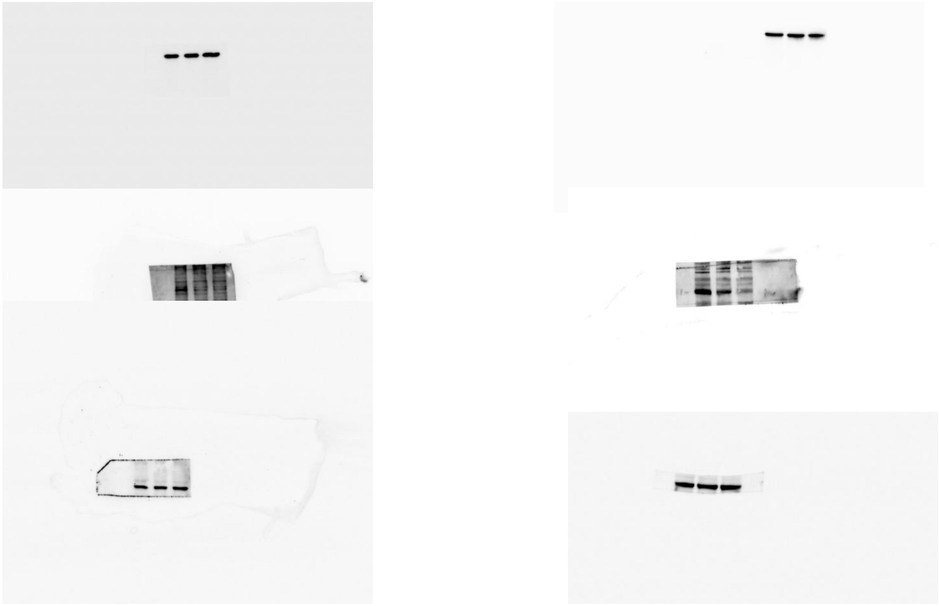

Figure 5E - ECA109

ANXA2

TTK

AKT

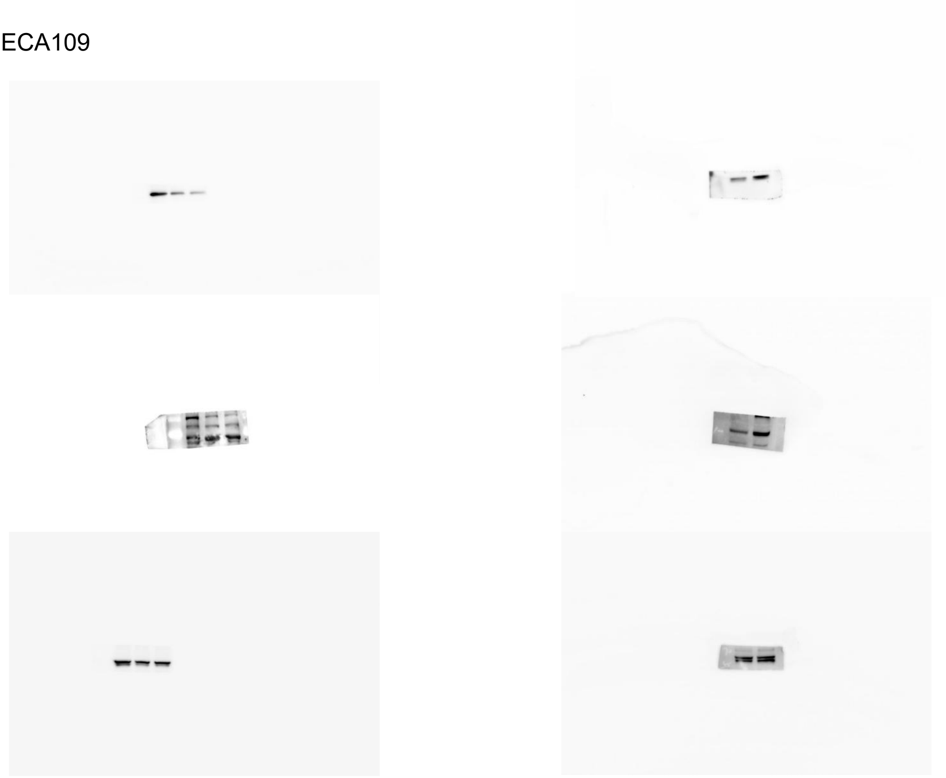

Figure 5E - ECA109

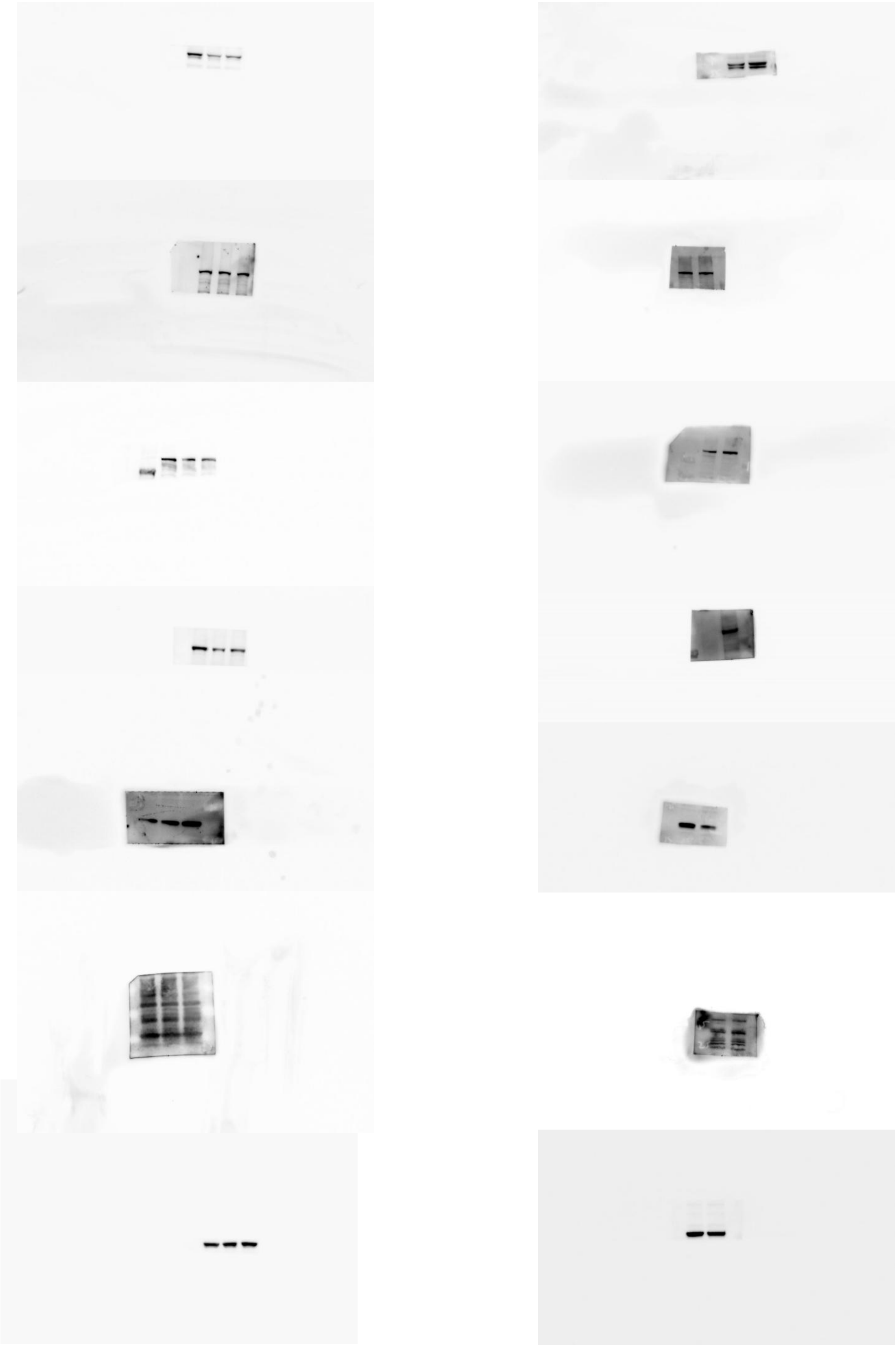

p-AKT^{S473}

MTOR

p-MTOR^{S2448}

β-catenin

Claudin-1

Snail

β-actin

Figure 5E - KYSE30

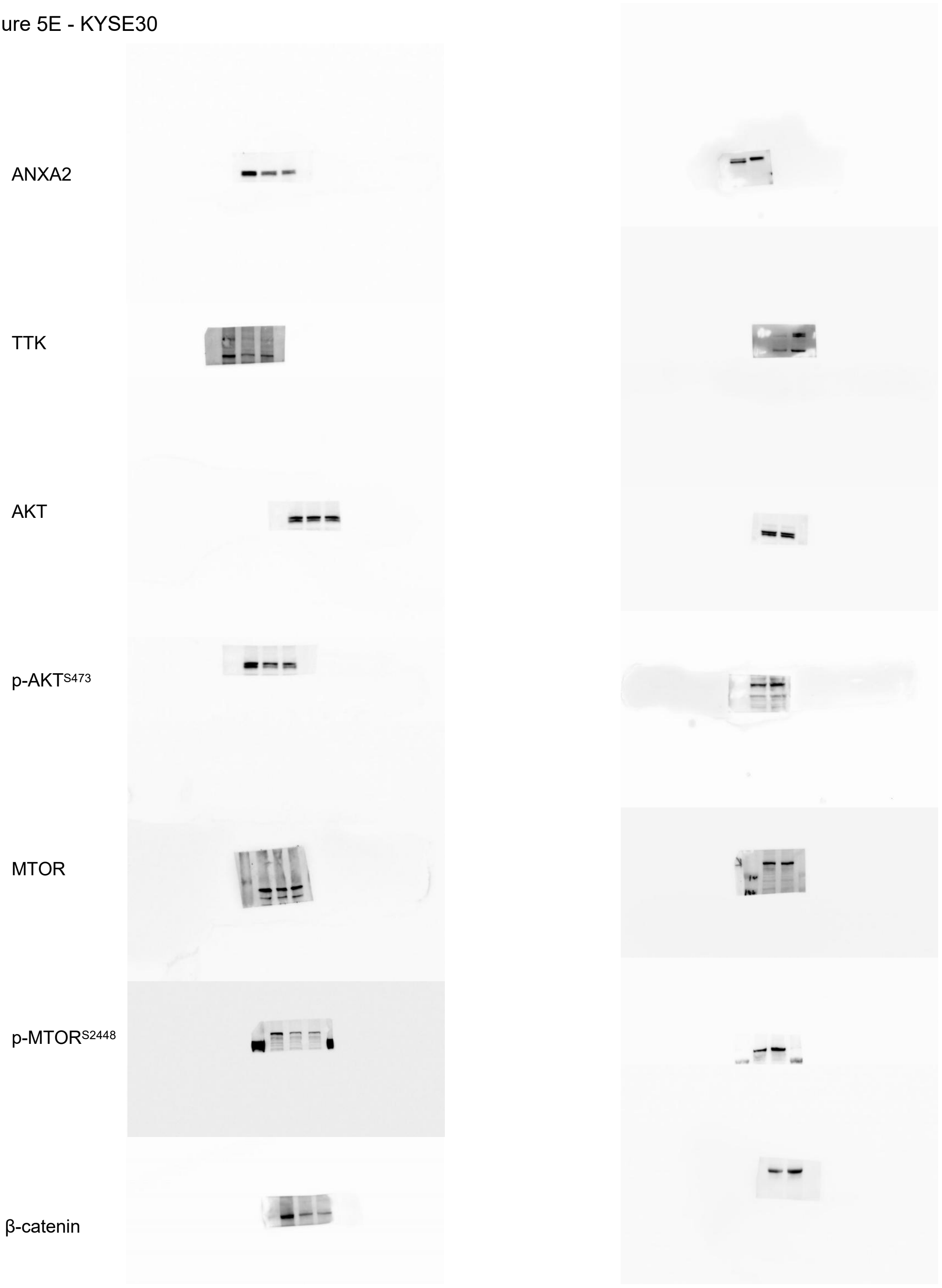

Figure 5E - KYSE30

Claudin-1

Snail

β-actin

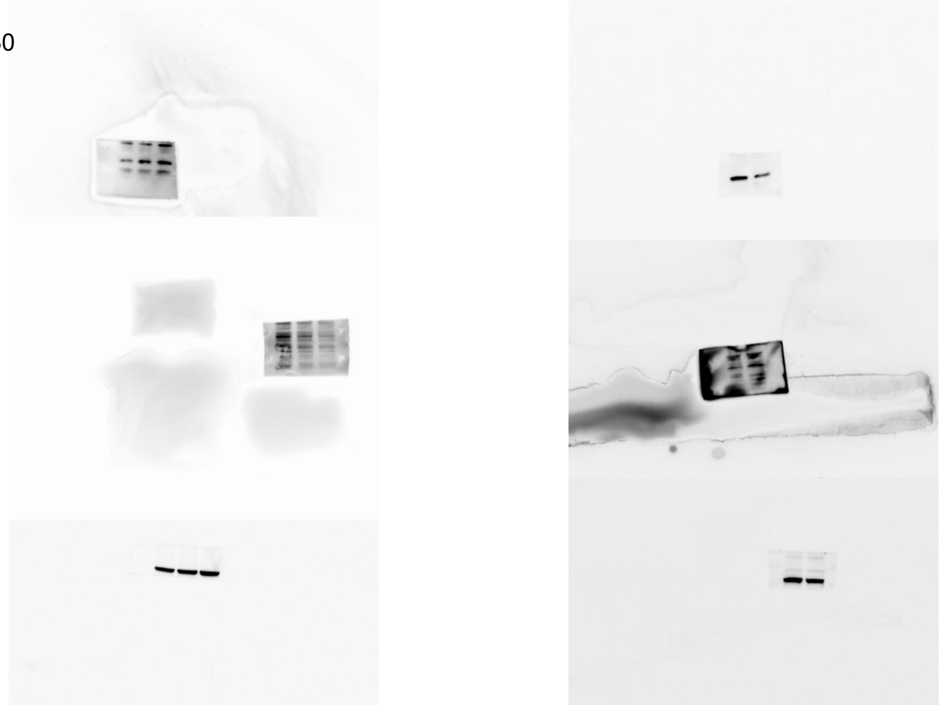

Figure 5F - ECA109

TTK

ANXA2

AKT

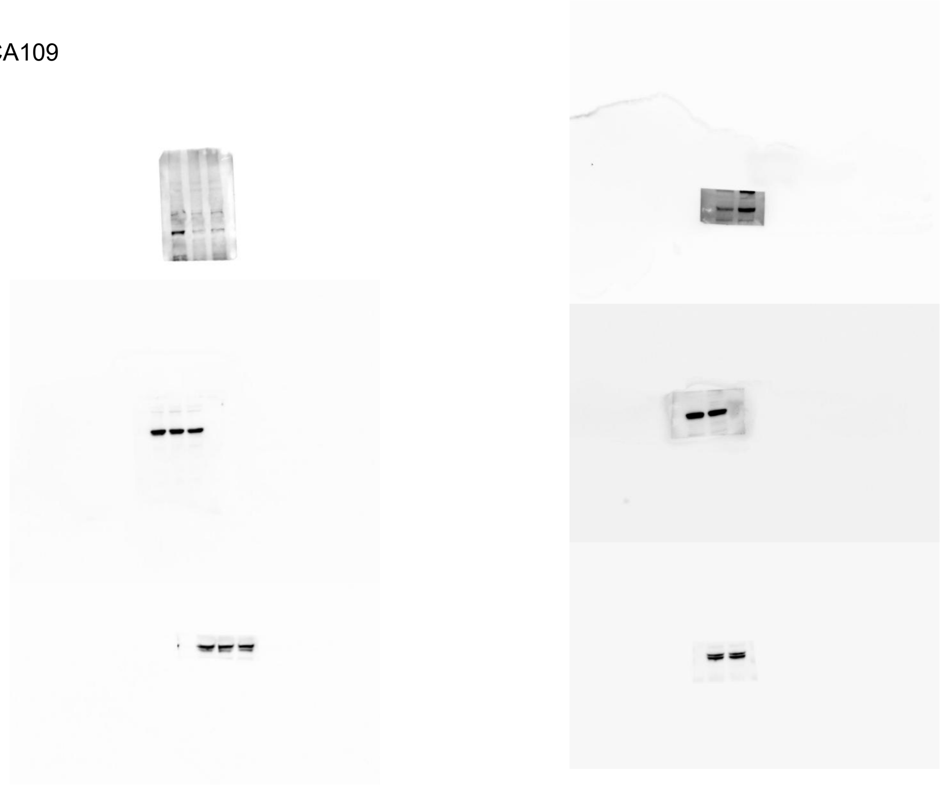

Figure 5F - ECA109

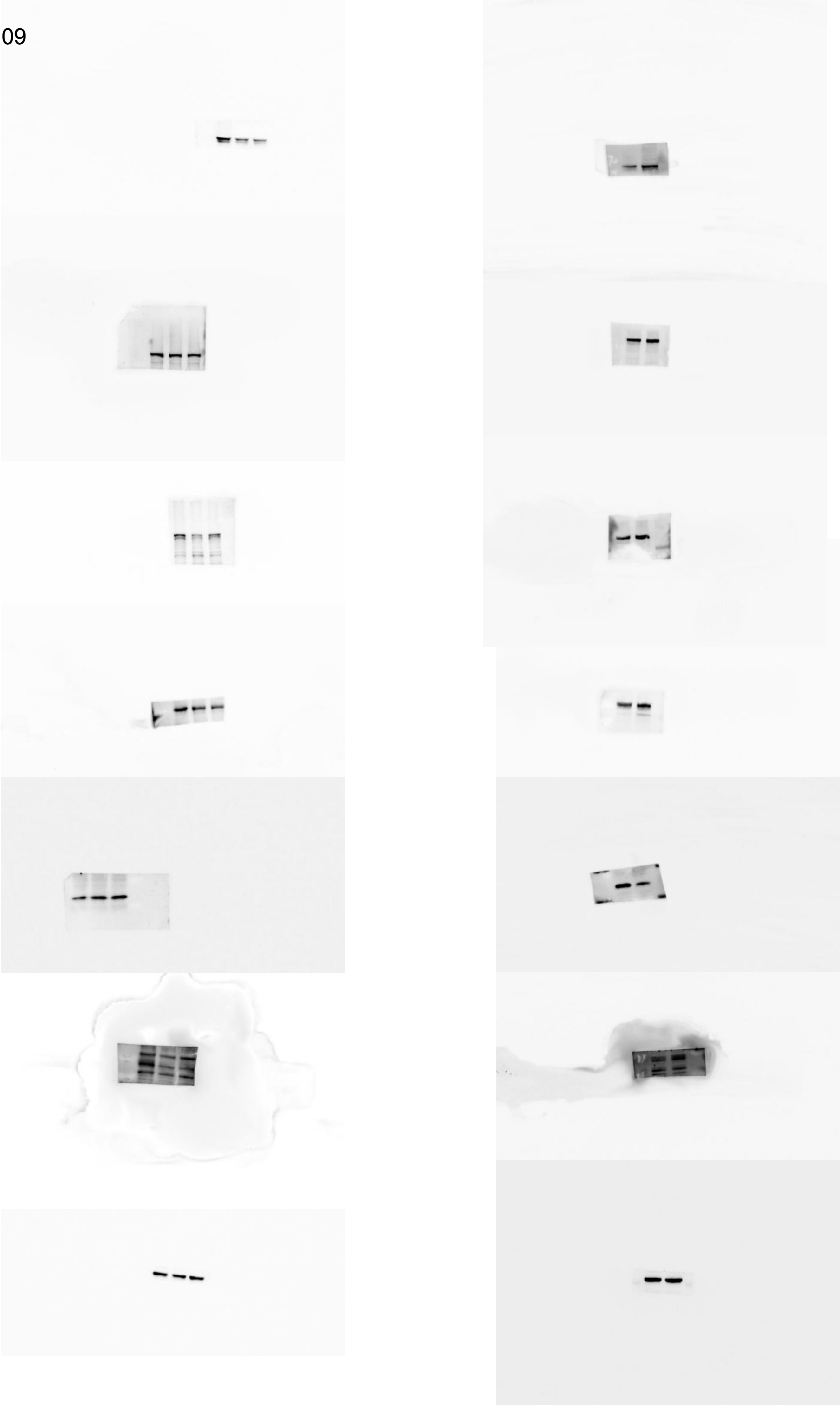

p-AKT^{S473}

MTOR

p-MTOR^{S2448}

β-catenin

Claudin-1

Snail

β-actin

Figure 5F - KYSE30

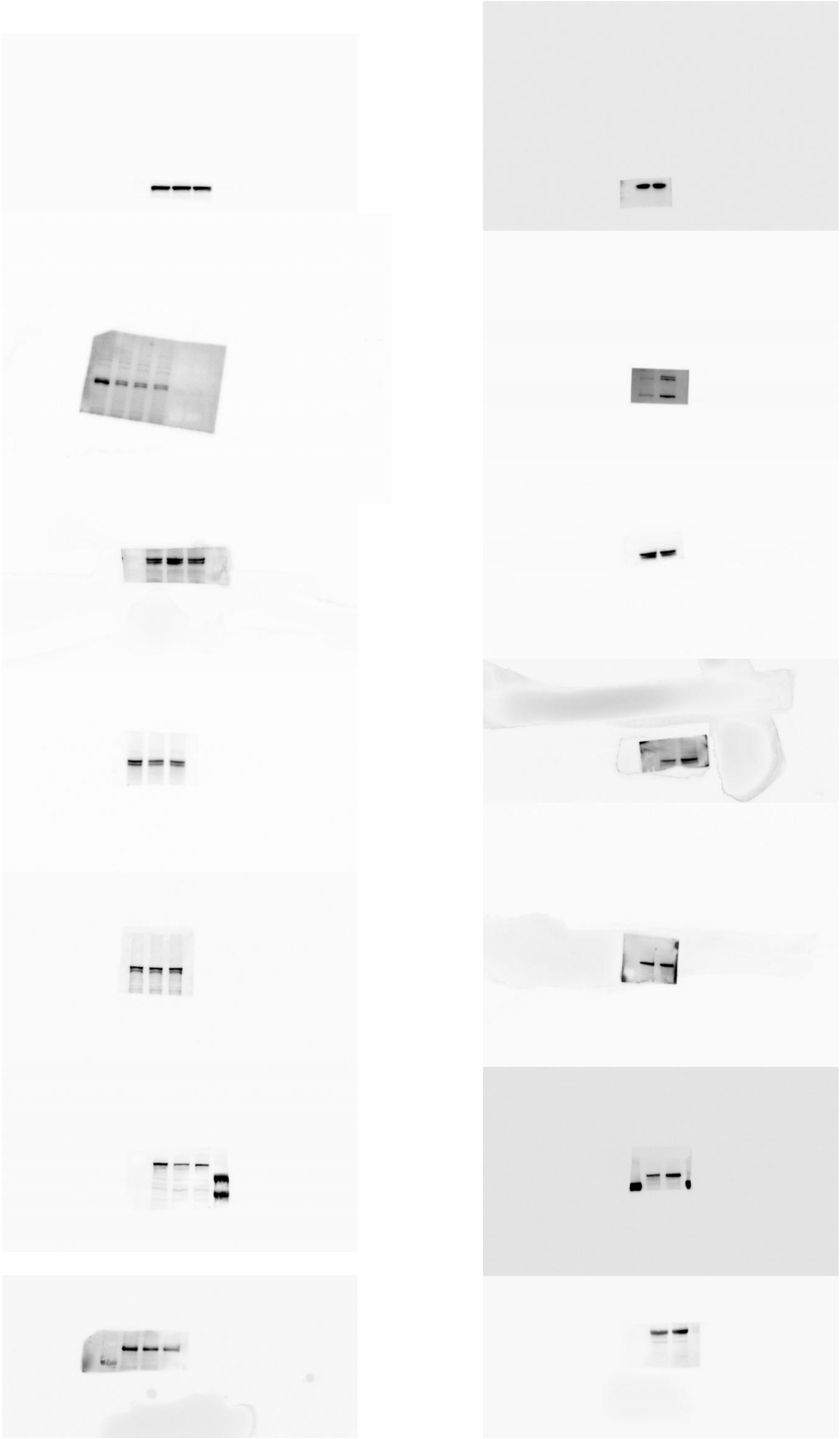

Figure 5F - KYSE30

Claudin-1

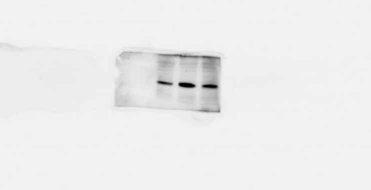

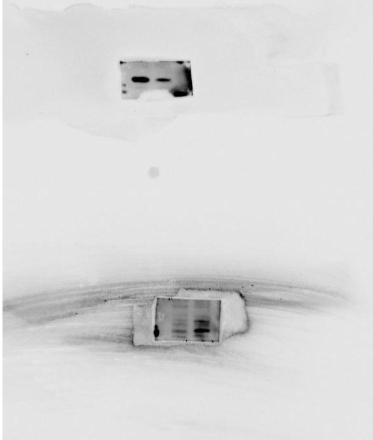

Snail

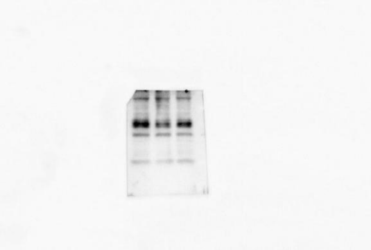

β-actin

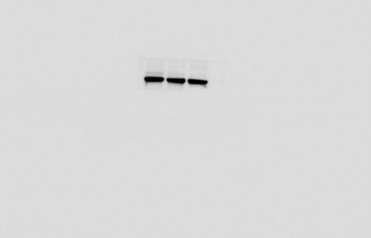

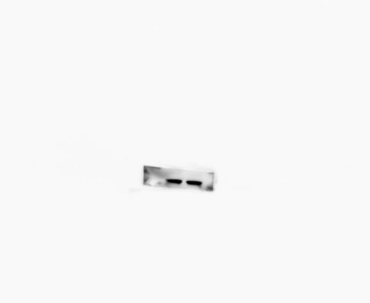

Figure 7A

ANXA2

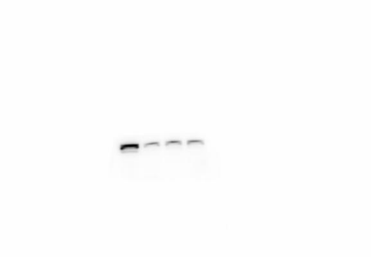

TTK

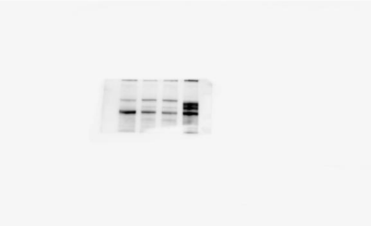

AKT

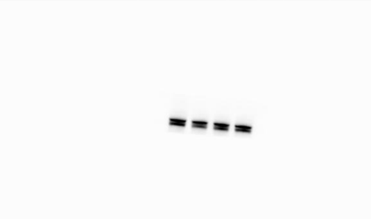

Figure 7A

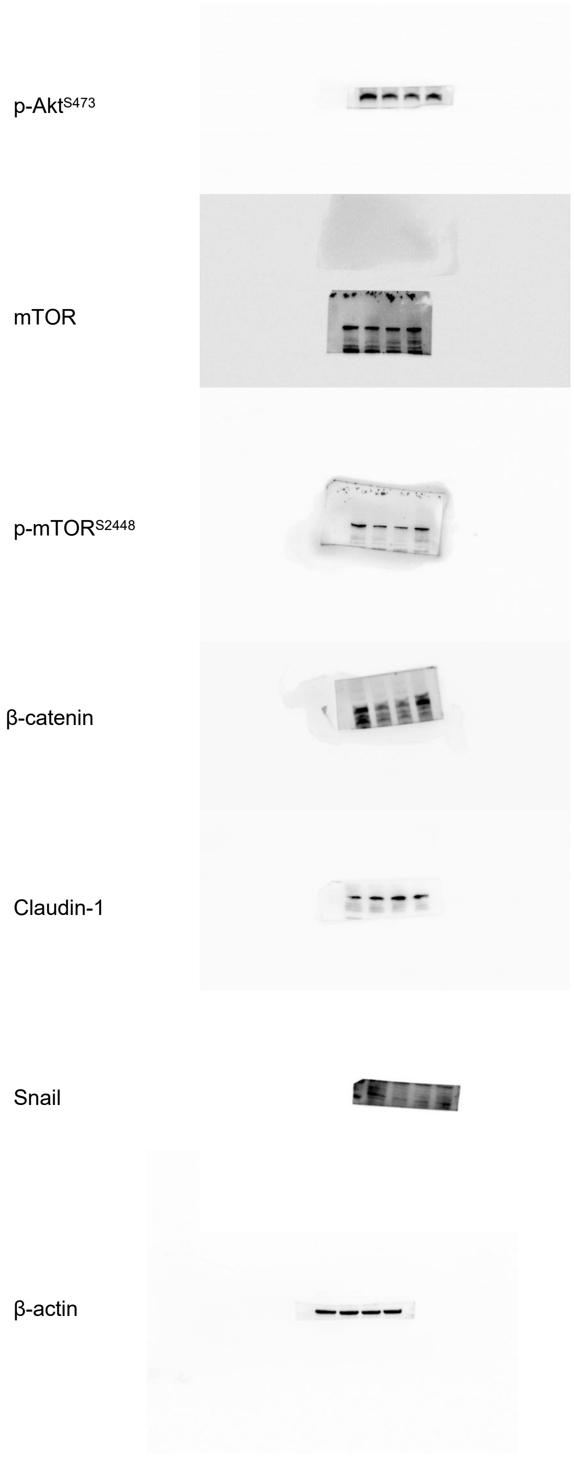

Figure 8A

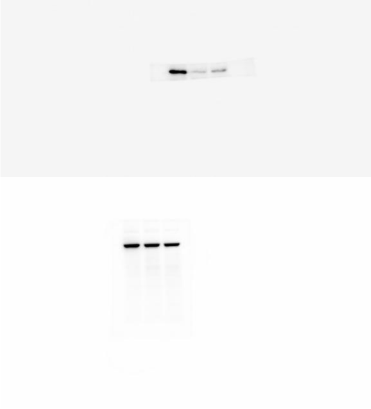

ANXA2

β-actin

Supplementary Figure 1J

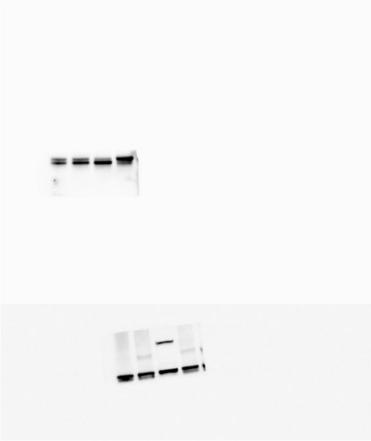

ANXA2

β-actin
